# Rivaroxaban versus warfarin to treat patients with thrombotic antiphospholipid syndrome, with or without systemic lupus erythematosus (RAPS): a randomised, controlled, open-label, phase 2/3, non-inferiority trial

**DOI:** 10.1016/S2352-3026(16)30079-5

**Published:** 2016-08-25

**Authors:** Hannah Cohen, Beverley J Hunt, Maria Efthymiou, Deepa R J Arachchillage, Ian J Mackie, Simon Clawson, Yvonne Sylvestre, Samuel J Machin, Maria L Bertolaccini, Maria Ruiz-Castellano, Nicola Muirhead, Caroline J Doré, Munther Khamashta, David A Isenberg

**Affiliations:** a Department of Haematology, University College London Hospitals NHS Foundation Trust, London, UK; b Department of Rheumatology, University College London Hospitals NHS Foundation Trust, London, UK; c Haemostasis Research Unit, Department of Haematology, University College London, London, UK; d Comprehensive Clinical Trials Unit, University College London, London, UK; e Centre for Rheumatology, Division of Medicine, University College London, London, UK; f Department of Thrombosis and Haemophilia, Guy's and St Thomas' Hospitals NHS Foundation Trust, London, UK; g Lupus Research Unit, Guy's and St Thomas' Hospitals NHS Foundation Trust, London, UK; h Department of Haematology, King's College London, London, UK; i Academic Department of Vascular Surgery, Cardiovascular Division, Faculty of Life Sciences and Medicine, King's College London, London, UK; j Lupus Research Unit, Division of Women's Health, King's College London, London, UK

## Abstract

**Background:**

Rivaroxaban is established for the treatment and secondary prevention of venous thromboembolism, but whether it is useful in patients with antiphospholipid syndrome is uncertain.

**Methods:**

This randomised, controlled, open-label, phase 2/3, non-inferiority trial, done in two UK hospitals, included patients with antiphospholipid syndrome who were taking warfarin for previous venous thromboembolism, with a target international normalised ratio of 2·5. Patients were randomly assigned 1:1 to continue with warfarin or receive 20 mg oral rivaroxaban daily. Randomisation was done centrally, stratified by centre and patient type (with *vs* without systemic lupus erythematosus). The primary outcome was percentage change in endogenous thrombin potential (ETP) from randomisation to day 42, with non-inferiority set at less than 20% difference from warfarin in mean percentage change. Analysis was by modified intention to treat. Other thrombin generation parameters, thrombosis, and bleeding were also assessed. Treatment effect was measured as the ratio of rivaroxaban to warfarin for thrombin generation. This trial is registered with the ISRCTN registry, number ISRCTN68222801.

**Findings:**

Of 116 patients randomised between June 5, 2013, and Nov 11, 2014, 54 who received rivaroxaban and 56 who received warfarin were assessed. At day 42, ETP was higher in the rivaroxaban than in the warfarin group (geometric mean 1086 nmol/L per min, 95% CI 957–1233 *vs* 548, 484–621, treatment effect 2·0, 95% CI 1·7–2·4, p<0·0001). Peak thrombin generation was lower in the rivaroxaban group (56 nmol/L, 95% CI 47–66 *vs* 86 nmol/L, 72–102, treatment effect 0·6, 95% CI 0·5–0·8, p=0·0006). No thrombosis or major bleeding were seen. Serious adverse events occurred in four patients in each group.

**Interpretation:**

ETP for rivaroxaban did not reach the non-inferiority threshold, but as there was no increase in thrombotic risk compared with standard-intensity warfarin, this drug could be an effective and safe alternative in patients with antiphospholipid syndrome and previous venous thromboembolism.

**Funding:**

Arthritis Research UK, Comprehensive Clinical Trials Unit at UCL, LUPUS UK, Bayer, National Institute for Health Research Biomedical Research Centre.

## Introduction

Thrombotic antiphospholipid syndrome is a potentially fatal and devastating disorder. The mainstay for secondary prevention of venous thromboembolism is anticoagulation with warfarin or other vitamin K antagonists.^1^, ^2^ Approximately 15% of patients with systemic lupus erythematosus have thrombotic antiphospholipid syndrome, which severely worsens the outlook.^3^ Antiphospholipid syndrome is classified as a rare disease,^4^ but a systematic review suggests that antiphospholipid antibodies are present in 10% of patients with deep vein thrombosis,^5^ which suggests possible underdiagnosis of antiphospholipid syndrome. Appropriate management of thrombotic antiphospholipid syndrome is crucial to minimise its harmful effects.

Direct oral anticoagulants, including rivaroxaban,^6^ are licensed for the treatment and secondary prevention of venous thromboembolism and are established as therapeutic alternatives to low-molecular-weight heparins and vitamin K antagonists. Patients with antiphospholipid syndrome were probably included in phase 3 randomised controlled trials of direct oral anticoagulants, but, because antiphospholipid antibody status was not systematically documented in these trials, confirmation of the usefulness of direct oral anticoagulants in these patients is required.

Generation of thrombin via the tissue factor pathway is integral to the blood coagulation process. Markers of in-vivo coagulation activation provide information about an individual's thrombogenic potential,^7^ and their concentrations should be reduced after anticoagulation.^8^ Thrombin generation triggered by tissue factor, therefore, seems to be a relevant marker.^9^ Thrombin generation acts as a global measure of anticoagulation and can show the anticoagulant effects of warfarin and rivaroxaban despite these drugs having different modes of action on the coagulation mechanism. The thrombin generation curve is quantified in terms of the lag time, time to peak thrombin generation, peak thrombin generation, and endogenous thrombin potential (ETP), which is the area under the curve. Warfarin reduces the ETP by 30–50% of that before warfarin or that in normal controls.^10^, ^11^ Rivaroxaban inhibits thrombin generation in whole blood and platelet-rich plasma,^12^ and the ETP may be used as a measure of anticoagulation intensity.^13^, ^14^

**Research in context**

**Evidence before this study**

We searched MEDLINE and PubMed with the following phrases: “antiphospholipid syndrome”, “systemic lupus erythematosus”, “venous thromboembolism”, “new oral anticoagulants”, “novel oral anticoagulants”, “direct acting oral anticoagulants”, “direct inhibitors of coagulation”, “non-vitamin K antagonist oral anticoagulants”, “warfarin”, “coumadin”, “vitamin K antagonists”, “dabigatran”, “rivaroxaban”, “apixaban”, “edoxaban”, “thrombin generation”, and “in vivo coagulation activation markers”. Further information was also requested from the manufacturers of the individual direct oral anticoagulants. Thrombotic antiphospholipid syndrome is a potentially fatal and devastating disorder. Although the disorder is rare, antiphospholipid antibodies are thought to be present in 10% of patients with deep vein thrombosis, suggesting possible underdiagnosis of thrombotic antiphospholipid syndrome. Warfarin and other vitamin K antagonists are the standard of care for the secondary prevention of venous thromboembolism in patients with thrombotic antiphospholipid syndrome. These drugs, however, can be particularly problematic in patients with thrombotic antiphospholipid syndrome because of variable sensitivity of thromboplastins to lupus anticoagulant, which is present in many of these patients. Consequently, the international normalised ratio (INR), which is used to monitor warfarin treatment, might not accurately reflect anticoagulation intensity. Two randomised controlled trials in thrombotic antiphospholipid syndrome have reported no benefits with high-intensity versus standard-intensity warfarin in the prevention of recurrent thrombosis. Rivaroxaban and other direct oral anticoagulants are effective and safe compared with warfarin for the treatment and secondary prevention of venous thromboembolism. Although antiphospholipid antibody status was not systematically documented in randomised controlled trials of direct oral anticoagulants, it is likely that patients with antiphospholipid syndrome were included. In the Rivaroxaban in Antiphospholipid Syndrome (RAPS) non-inferiority trial, therefore, we aimed to confirm the usefulness of rivaroxaban in secondary prevention of venous thromboembolism in patients with antiphospholipid syndrome.

**Added value of this study**

RAPS is slightly larger than the two previous randomised controlled trials in patient with thrombotic antiphospholipid syndrome, and our inclusion criteria enabled recruitment of a more homogeneous study population, that is, only patients with previous venous thromboembolism needing standard-intensity warfarin and none with arterial thrombosis related to antiphospholipid syndrome, which is not a licensed indication for direct oral anticoagulants. Because thrombotic antiphospholipid syndrome is clinically heterogeneous, the homogeneity of our study population maximises the clinical applicability of our results. Thrombin generation allows assessment of the anticoagulant effects of warfarin and rivaroxaban despite these drugs' different modes of action. When assessed by endogenous thrombin potential ([ETP] ie, the are under curve) alone, rivaroxaban was inferior to warfarin in terms of anticoagulation intensity, but peak thrombin generation favoured rivaroxaban. Warfarin affects all thrombin generation parameters equally, whereas rivaroxaban mainly affects the initiation and propagation phases of thrombin generation. Formation of the prothrombinase complex is delayed and lag time and time to peak thrombin generation are prolonged and, therefore, the ETP is greater than would be expected for the degree of anticoagulation. Thus, the overall thrombogram indicated no increase in thrombotic risk with rivaroxaban. This conclusion was supported by concentrations of in-vivo coagulation activation markers being increased in only a few patients in both treatment groups, and the absence of new thrombotic events during 6 months of treatment. No major bleeding episodes were noted, and rivaroxaban was significantly associated with improved quality of life. Additionally, we found no evidence in in-vitro studies of antiphospholipid antibodies interfering with the anticoagulant action of rivaroxaban.

**Implications of all the available evidence**

Rivaroxaban seems to offer an effective, safe, and convenient alternative to warfarin in patients with thrombotic antiphospholipid syndrome who have had previous venous thromboembolism requiring standard-intensity warfarin therapy (ie, target INR 2·5, range 2·0–3·0). The RAPS findings are applicable to this group of patients due to the homogeneity of the study population. An alternative to warfarin would be welcomed by these patients and their treating clinicians, particularly because of issues with variable sensitivity of thromboplastins to lupus anticoagulant and unstable INR needing frequent and unpredictable anticoagulant monitoring. Warfarin is also associated with risks of thrombosis or bleeding due to underanticoagulation and overanticoagulation, respectively. The RAPS trial, however, was designed with a laboratory surrogate outcome measure that reflects the mechanisms of action of the interventions because the large-scale, long-term clinical trials needed to assess recurrent venous thromboembolism are not feasible in patients with antiphospholipid syndrome. The absence of new thrombosis or major bleeding and the low rate of clinically relevant bleeding indicate low risks in the subgroup of patients assessed and puts into context anecdotal reports in case studies and small case series of recurrent thrombosis after switching from warfarin to a direct oral anticoagulant in patients with antiphospholipid syndrome. The small but significant improvement in the quality of life visual analoge score seen with rivaroxaban in RAPS is encouraging. Further studies are needed to define the role of direct oral anticoagulants in the treatment of patients with antiphospholipid syndrome, including those who need higher-intensity anticoagulation after recurrent thrombotic events while they were taking standard-intensity anticoagulation, and those with stroke or other arterial thrombosis.

We did the Rivaroxaban in Antiphospholipid Syndrome (RAPS) trial to investigate whether rivaroxaban would provide anticoagulation non-inferior to that achieved with standard-intensity warfarin in patients with antiphospholipid syndrome and previous venous thromboembolism, with or without systemic lupus erythematosus. The study protocol has been published^15^ and is available online.

## Methods

### Study design

RAPS was a randomised, controlled, open-label, phase 2/3, non-inferiority clinical trial in patients with thrombotic antiphospholipid syndrome who were receiving standard-intensity warfarin for venous thromboembolism (appendix p 3). We recruited patients from specialist haematology and rheumatology clinics at University College London Hospitals and Guy's and St Thomas' Hospitals NHS Foundation Trusts, London, UK. Enrolled patients provided informed written consent after discussion with a hospital study investigator or a delegate.

The trial was overseen by an independent trial steering committee (appendix p 5). An independent data monitoring committee (appendix p 5) provided oversight and monitored trial progress. Ethics approval was obtained from the University College London Hospitals NHS Foundation Trust research and development office, having been approved by the National Research Ethics Service Committee South Central-Oxford A (reference 12/SC/0566).

### Patients

Eligible patients had thrombotic antiphospholipid syndrome (appendix p 3)^16^ and at least one venous thromboembolism when taking no or subtherapeutic anticoagulant therapy (appendix p 3), and had been taking standard-intensity warfarin (target international normalised ratio [INR] 2·5) for at least 3 months since the last venous thromboembolic event. Women had to be using adequate contraception (appendix p 3) unless they were postmenopausal or had undergone sterilisation.

We excluded patients with previous arterial thrombotic events (appendix pp 3, 4) due to antiphospholipid syndrome or recurrent venous thromboembolism when taking warfarin at a therapeutic INR of 2·0–3·0 and those who were younger than 18 years. Other exclusion criteria were pregnancy or lactation; severe renal impairment (creatinine clearance calculated with the Cockcroft and Gault formula^17^, ^18^ ≤29 mL/min); alanine aminotransferase more than twice the upper limit of normal; Child-Pugh class B or C cirrhosis; thrombocytopenia (platelets <75 × 10^9^/L); non-adherence to warfarin regimen (based on clinical assessment); taking azole class antifungals, protease inhibitors (eg, ritonavir) for HIV, strong CYP3A4 inducers (eg, rifampicin, phenytoin, carbamazepine, phenobarbital, or St John's wort), or dronedarone; and refusal to give consent for the study site to inform a family doctor or health-care professional responsible for anticoagulation care about participation.

All patients enrolled met the international consensus criteria for antiphospholipid syndrome,^16^ with testing for antiphospholipid antibodies done in accordance with national and international guidelines (appendix pp 6, 7).^16^, ^19^, ^20^ All patients with systemic lupus erythematosus were classified according to the revised criteria of the American College of Rheumatologists^21^ and reviewed in lupus clinics by experienced clinicians, according to standard activity and damage assessment indices, although the results were not part of this study.

We did not apply any performance status criteria for trial entry, and all patients included in the trial were outpatients. We did not anticipate that mortality during follow-up would differ from that in the general population.

### Randomisation and masking

Randomisation was performed by a web-based independent randomisation service (Sealed Envelope, London, UK) to ensure allocation concealment. The schedule was created using permuted blocks with a random block length, stratified by centre and patient type (with *vs* without systemic lupus erythematosus). Participants were randomised 1:1 to remain on standard-intensity warfarin with target INR 2·5 (range 2·0–3·0) or to switch to 20 mg oral rivaroxaban once daily (or 15 mg once daily depending on local clinical care and following the summary of product characteristics in patients with creatinine clearance 30–49 mL/min) for 180 days (appendix pp 8, 9).^6^

The trial was open label to ensure optimum warfarin dosing, as the variable sensitivity of thromboplastins to lupus anticoagulant^22^ can lead to INR instability. This and other factors, such as changing medication, can necessitate frequent anticoagulant monitoring with unpredictable time intervals. Additionally, the management of bleeding events differs between patients receiving warfarin and rivaroxaban. Masking of treatment allocation was also not possible in the RAPS central laboratory because different tests were needed for the two anticoagulants, and samples taken at baseline and day 42 were tested simultaneously to minimise variability between assays.

### Follow-up

Trial follow-up continued for 210 days. Patients or clinicians could choose to stop treatment early because of unacceptable serious adverse events (SAEs), thrombotic events, any change in the patient's condition that justified discontinuation (decided by clinician; included needing any drug specified in the exclusion criteria), withdrawal of consent (decided by patient), and pregnancy.

In patients with renal impairment, the dose of rivaroxaban could be modified if creatinine clearance changed. Patients receiving 20 mg rivaroxaban once daily could receive 15 mg if creatinine clearance changed to 30–49 mL/min,^6^ and in patients receiving 15 mg daily, the dose could be changed to 20 mg if creatinine clearance changed to 50 mL/min or more. Treatment with rivaroxaban could also be temporarily stopped if a patient had a bleeding event or needed bridging anticoagulation for a procedure (routine or emergency).

### Assessments

Venous blood was collected at baseline and day 42 with minimum venostasis, into 0·105 M citrate Vacutainer tubes (BD, Plymouth, UK). Platelet-poor plasma was prepared within 2 h by double centrifugation (2000 g for 15 min at ambient temperature) and stored at −80°C. Trial assays were performed in the RAPS central laboratory in the Haemostasis Research Unit, University College London, London, UK. Patients taking rivaroxaban were asked to attend for venepuncture on day 42 2–4 h after the rivaroxaban dose to capture the peak for assessment of thrombin generation and rivaroxaban anti-Xa levels.

Thrombin generation testing was done with the Calibrated Automated Thrombogram^9^ and PPP Reagent (Diagnostica Stago, Asnières sur Seine, France).^23^ ETP and peak thrombin generation results were normalised by use of thrombin generation test reference plasma (National Institute for Biological Standards, Potters Bar, UK) to reduce interassay variability.^24^ The intra-assay and interassay coefficients of variation for lag time, ETP, and peak thrombin generation were 0·8–1·4% and 2·0–2·6%, respectively.

To assess in-vivo coagulation activation, we measured prothrombin fragment (F1.2), thrombin–antithrombin complex, and D-dimer concentrations.^23^

Prothrombin time was assessed with PT-Fibrinogen HS Plus on a TOP500 (Werfen, Warrington, UK) with an analyser-specific international sensitivity index. INR monitoring of patients assigned to continue warfarin was done in their usual anticoagulation clinics. Factor X activity was measured with an amidolytic assay (Hyphen Biomed, Neuville-Sur-Oise, France) on the CS-2000*i* analyser (Sysmex UK, Milton Keynes, UK).^25^ A previously established therapeutic range for amidolytic factor X of 18–33 IU/dL, which corresponds to INR 2·0–3·0, was used to assess anticoagulation intensity.^25^ The intra-assay coefficient of variation with normal plasma was 8·3%.

Rivaroxaban concentrations were measured with the Biophen DiXaI amidolytic anti-Xa assay (Hyphen BioMed) on the CS-2000*i* analyser.^23^ Intra-assay coefficients of variation were 1·3% at 300 μg/L and 8·0% at 100 μg/L.

Antiphospholipid antibody status was assessed by the RAPS central laboratory at baseline, in accordance with national and international guidelines.^16^, ^19^, ^20^ Lupus anticoagulant was assessed by the dilute Russell's viper venom time, using Siemens Healthcare (Marburg, Germany) LA1 (screening) and LA2 (confirmation) reagents, and the Taipan venom time-to-ecarin clotting time ratio (Diagnostic Reagents, Thame, UK). The normalised ratio cutoff value for both tests was 1·2. IgG or IgM antibodies against cardiolipin and β_2_ glycoprotein I (β_2_GPI) were measured with Quanta Lite kits (Inova Diagnostics, San Diego, CA, USA). Moderate to high positivity for antibodies against cardiolipin was defined as greater than the 99th centile (ie, >20 GPLU or MPLU) and for antibodies against β_2_GPI positivity as greater than the 99th centile (ie, >20 SGu or SMu). Triple positivity was defined as concentrations of antibodies against cardiolipin and β_2_GPI greater than the 99th centile and a positive test for lupus anticoagulant, in accordance with the international consensus criteria.^16^

### Safety

Reports of SAEs, serious adverse reactions, and suspected unexpected serious adverse reactions were reviewed by external, independent, medically qualified staff. SAEs were graded according to the Common Terminology Criteria for Adverse Events (version 4.0). Clinically relevant and minor bleeding events across all sites were pseudoanonymised and reviewed by one investigator (DAI) to remove the potential bias of interoperator variation. The classification of bleeding events as clinically relevant or minor, as per the protocol (appendix p 10), was checked and changed if appropriate. Product-related non-serious adverse events were to be reported if deemed by the investigator to have occurred due to the drug not working (appendix p 4).

### Outcome measures

The primary outcome was the percentage change in ETP from randomisation to day 42 (first trial visit after randomisation). Intensity of anticoagulation was assessed with thrombin generation. We chose the first visit for assessment because the pharmacokinetics of rivaroxaban suggest that the therapeutic effect would be stable after a few days of treatment (the protocol specified that rivaroxaban treatment must begin within 10 days of randomisation) and there would be no residual effect from warfarin on the ETP because the maximum biological half-life of the vitamin-K-dependent coagulation factors is 72 h.^26^

Secondary outcomes for efficacy were the occurrence of thromboembolism up to day 210 after randomisation, whether these were venous thromboembolism alone or a composite of venous thromboembolism and other thrombotic events (appendix pp 3, 4), thrombin generation (lag time, time to peak thrombin generation, peak thrombin generation, and ETP) at baseline and on day 42, and markers of in-vivo coagulation activation at baseline and day 42. Secondary outcomes for safety were SAEs and bleeding events from baseline to day 210. Other secondary outcomes were adherence to treatment, assessed by laboratory testing of INR and amidolytic factor X for warfarin and anti-factor Xa rivaroxaban level for rivaroxaban, both at day 42; percentage of time between baseline and day 180 in the therapeutic range for warfarin; and quality of life, assessed with the five-level version of EuroQol-5D (EQ-5D-5L) at baseline and day 42.

### Statistical analysis

We set the threshold for non-inferiority of rivaroxaban for the primary outcome at less than 20% difference from warfarin in the mean percentage change. This limit was based on variability of test performance between centres^27^ and clinical relevance. We calculated that if there were truly no difference between groups in the mean percentage change in ETP, we would need to enrol 58 patients per group to ensure with 80% power that a two-sided 95% CI would exclude the non-inferiority threshold, assuming a common SD of 36%, one-sided significance level of 2·5%, and 12% of patients who were not assessable for the primary outcome.

Analyses were done according to a prespecified statistical analysis plan (appendix pp 11–21) except for an exploratory post-hoc subgroup analysis for interactions between the effects of rivaroxaban and lupus anticoagulant positivity at baseline for any thrombin generation parameter (appendix pp 22, 23). We used a modified intention-to-treat approach to include all randomised patients with assessable data in all analyses. Descriptive statistics were used to summarise patients' demographic, clinical, and other outcomes. We assessed the primary outcome with a regression model to estimate the difference in log-transformed ETP between rivaroxaban and warfarin at day 42, with a two-sided 95% CI, adjusted for stratification variables and baseline ETP. Estimates and 95% CIs on the log scale were back-transformed to the original scale (appendix p 24). This approach was also used to analyse differences between treatment groups for secondary thrombin generation parameters (lag time, time to peak thrombin generation, and peak thrombin generation) in-vivo coagulation activation markers, and EQ-5D-5L. Fisher's exact tests were used to compare proportions. Pearson's correlation coefficient, or Spearman's rank correlation coefficient were used to explore relationships between ETP, INR, and laboratory measures of adherence.

Values lower than the lower limits of detection for thrombin generation parameters and rivaroxaban concentrations (ie, censored values) were excluded from the analysis because they cannot be handled in linear regression models. Patients providing non-censored samples were not systematically different from those who did not and, therefore, we judged it was reasonable to assume that these were missing completely at random. Because the proportion of incomplete data (censored and missing values) for each outcome was small (5%), we did no imputations.

Two sensitivity analyses were planned for the primary outcome: a per-protocol analysis, as is recommended for non-inferiority trials, and tobit regression analysis to account for censored values (ie, those outside the assay limit of detection). However, neither was required as all patients were still taking their allocated treatments on day 42, and only one patient with censored values in the primary outcome had non-censored baseline data that could have contributed to the sensitivity analysis (appendix p 26).

All statistical analyses were done with Stata/IC version 13.1. This trial is registered with the ISRCTN registry, number ISRCTN68222801.

### Role of the funding source

Except for the University College London, which, represented by the Comprehensive Clinical Trials Unit at UCL by formal delegated authority, undertook the RAPS trial as a development project, none of the funders had involvement in the study design, data collection, data analysis, data interpretation, writing of the report, or decision to submit for publication. The corresponding author had full access to all the data in the study and had the final responsibility for the decision to submit for publication.

## Results

**Figure 1:**
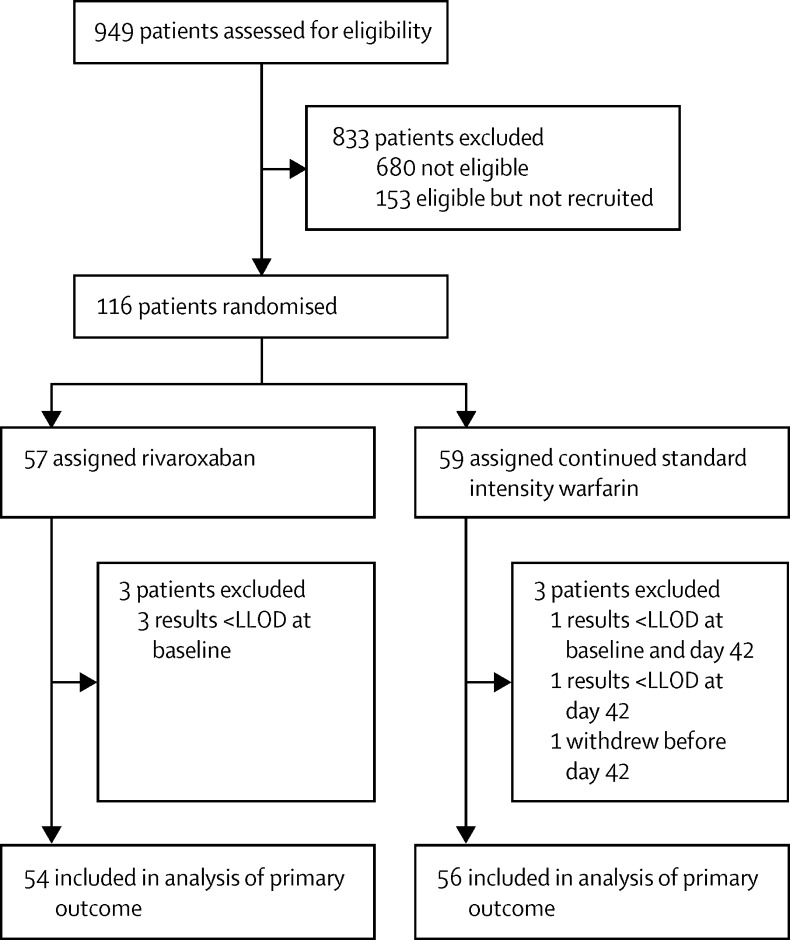
Trial profile LLOD=lower limit of detection.

116 patients were recruited between June 5, 2013, and Nov 11, 2014 (figure 1). The final day 42 visit, when laboratory outcomes were assessed, was on Dec 22, 2014, and the final day 210 visit, when clinical outcomes were assessed, was on June 8, 2015. 57 patients were assigned to receive rivaroxaban and 59 to receive warfarin, and all patients received their allocated treatments. Of these 116 patients, six (5%) did not contribute data for the primary outcome. Therefore, the primary analysis population included 110 patients (54 in the rivaroxaban group and 56 in the warfarin group). Baseline characteristics were similar in the two groups (table 1). 11 patients in both groups had systemic lupus erythematosus. Four (3%) of 116 patients had other autoimmune rheumatic disorders. Numbers of withdrawals, losses to follow-up, and missing outcome data, and the number and proportion of cases excluded from the analyses by outcome measure and treatment group are shown in the appendix (pp 25–27). Measures of treatment exposure for the 113 patients that completed the trial treatment visits (day 180) are also shown (appendix pp 28, 29).

**Figure 2:**
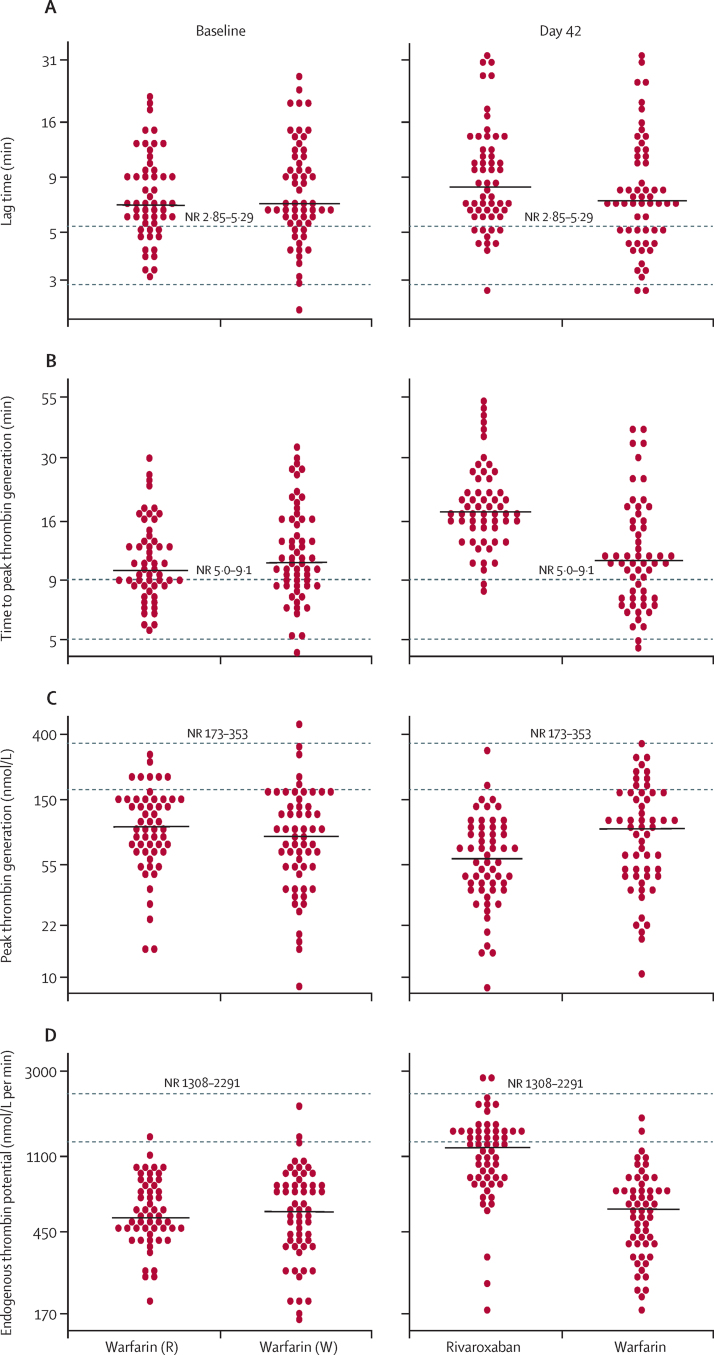
Thrombin generation parameters at baseline (left) and day 42 (right) Solid lines indicate medians, dotted lines indicate limits of normal ranges. NR=normal range. Warfarin (W)=patients receiving warfarin at baseline who continued taking warfarin after randomisation. Warfarin (R)=patients receiving warfarin at baseline who were switched to rivaroxaban at randomisation.

**Figure 3:**
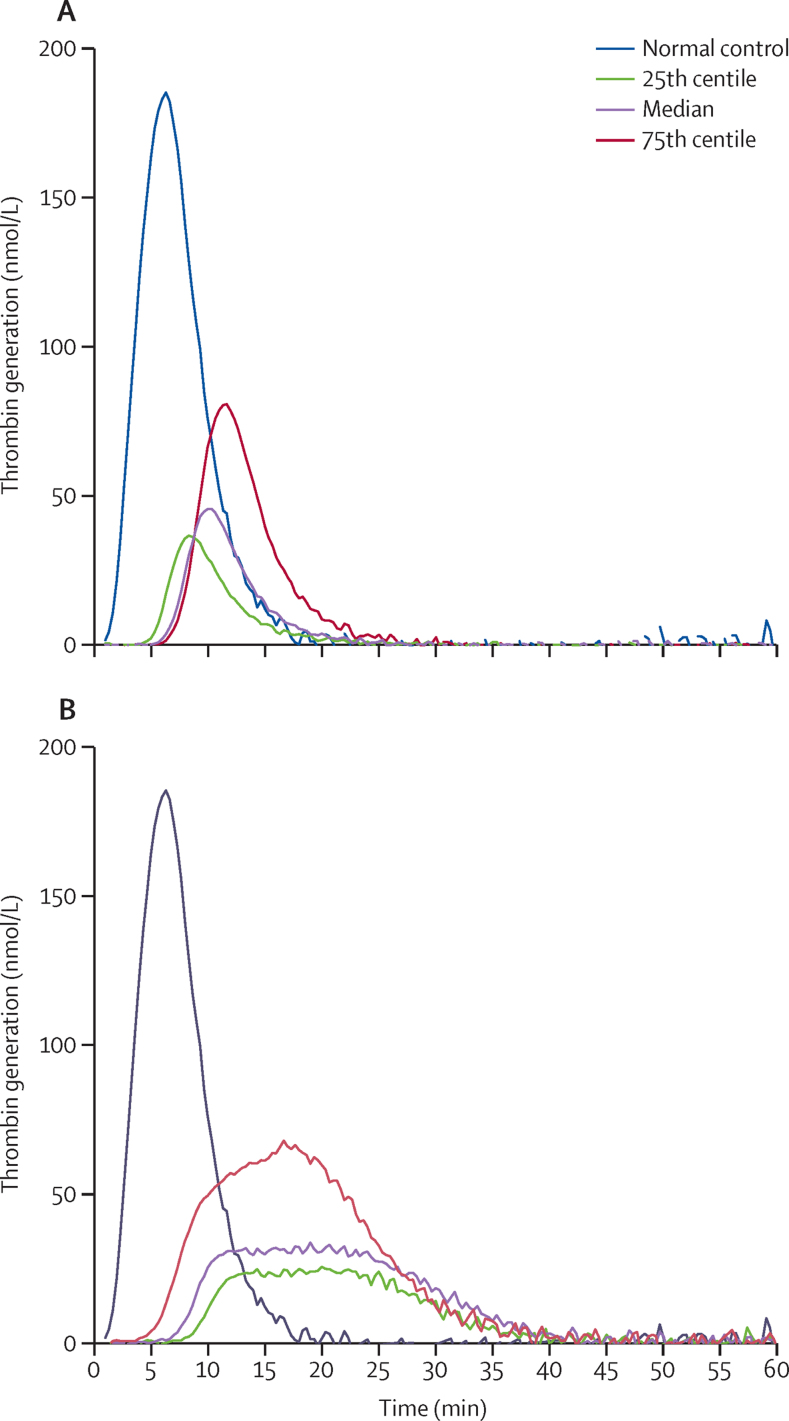
Thrombograms for median (25th and 7th percentiles) ETP values in RAPS, compared with a typical normal control value (A) Patients taking warfarin. (B) Patients taking rivaroxaban. ETP=endogenous thrombin potential. RAPS=the Rivaroxaban in Antiphospholipid Syndrome trial.

Thrombin generation parameters in the two groups were similar at baseline. At day 42, ETP was significantly higher in the rivaroxaban group than in the warfarin group (table 2, figure 2). The mean percentage change in ETP did not reach the non-inferiority threshold. By contrast, lag time and time to peak thrombin generation were significantly longer and the peak thrombin generation was significantly lower in patients taking rivaroxaban (table 2, figure 2). Examples of typical RAPS thrombograms are shown in figure 3. The exploratory post-hoc subgroup analysis showed no significant interactions between the effects of rivaroxaban and lupus anticoagulant positivity at baseline on thrombin generation (appendix pp 22, 23).

Concentrations of F1.2, thrombin–antithrombin complex, D-dimer, or a combination of these, at day 42 were raised above the normal range in three (5%) of 57 patients taking rivaroxaban and six (10%) of 58 taking warfarin. Of these, one and two, respectively, also had raised in-vivo coagulation activation markers at baseline.

Peak rivaroxaban concentrations in plasma at day 42 were at least 160 μg/L in 29 (51%) of 57 patients (>360 μg/L in three) and correlated negatively with ETP (*r*_s_=–0·5, 95% CI −0·7 to −0·2). Among the 28 patients with concentrations lower than 160 μg/L, eight were between 50 and 100 μg/L, and six were lower than the lower limit of detection of 50 μg/L. Blood samples for measurement were taken at 2–4 h after treatment in 39 (70%) of 56 patients, and within 6 h in all except four patients (range 8–24 h).

Amidolytic factor X in patients taking warfarin correlated positively with ETP at day 42 (*r*=0·5, 95% CI 0·3–0·7). Correlations between INR and ETP were negative in the rivaroxaban and warfarin groups at baseline, and for the warfarin group at day 42 (*r*=–0·5, 95% CI −0·7 to −0·3 at both baseline and day 42). The percentage of time in the therapeutic range for patients taking warfarin was similar at baseline (table 1) and day 180 (table 2).

No thrombotic events were seen in patients in either group during 6 months of taking treatment. No patients required dose reductions or discontinuation of the allocated intervention because of drug-related toxic effects. No major bleeding events were reported in either group up to day 210 (table 2). The numbers of other safety events (SAEs and clinically relevant or minor bleeding events) did not differ between groups.

Four SAEs were reported in patients taking rivaroxaban. Two were judged to be unrelated to the trial drug. The first was an intracranial haemorrhage that pre-dated the trial and was detected incidentally on brain imaging without any clinical or imaging indications of new or recurrent bleeding (grade 1). The second was an episode of abdominal pain, vomiting, arthralgia, and myalgia (grade 2). The other two SAEs were deemed unlikely to be related to the trial drug. The first of these was a suspected deep vein thrombosis at day 176, identified after the patient presented with leg pain and swelling on a background of chronic post-thrombotic lower limb swelling following a previous femoral deep vein thrombosis. A lower limb venous doppler scan showed changes related to the previous femoral vein deep vein thrombosis but no new thrombosis. Rivaroboxan was stopped while the patient received treatment with therapeutic dose low-molecular-weight heparin, then restarted (grade 2). This episode was reported as an SAE because of the potential seriousness of the situation. The second of these SAEs was intestinal perforation (grade 4).

Four SAEs were reported in patients taking warfarin, three of which were judged to be unrelated to the trial drug: one patient had an acute exacerbation of asthma associated with an upper respiratory tract infection (grade 3), one had sepsis (grade 4), and one developed high-grade non-Hodgkin lymphoma stage IVB and subsequently died. The fourth patient had clinically relevant haemorrhoidal haemorrhage that was deemed probably to have been related to warfarin (grade 3 severe adverse reaction). No suspected unexpected serious adverse reactions were reported. There were no treatment-related deaths.

EQ-5D-5L health utility scores did not differ between groups (mean difference 0·04, 95% CI −0·02 to 0·09, p=0·19; table 2). A small difference was seen between groups in the visual analogue health score, favouring the rivaroxaban group (mean difference 6·5, 95% CI 1·4–11·5, p=0·013).

## Discussion

When anticoagulation intensity was assessed by percentage change in ETP alone, rivaroxaban was inferior to warfarin in patients with antiphospholipid syndrome and previous venous thromboembolism. However, peak thrombin generation was lower with rivaroxaban and, therefore, the overall thrombogram indicated no difference in thrombotic risk. This conclusion is supported by in-vivo coagulation activation marker concentrations being raised in only a few patients in both treatment groups. Additionally, no new thrombotic events were seen during 6 months of treatment. No patients had major bleeds, and the frequencies of clinically relevant and minor bleeding were similar in the two groups. Quality of life, as measured with EQ-5D-5L visual analogue scores, was significantly better in the rivaroxaban group than in the warfarin group.

Rivaroxaban and warfarin both inhibit thrombin generation in patients with venous thromboembolism who do not have antiphospholipid syndrome,^23^ indicating effective anticoagulation. Inhibition of thrombin generation, which indicates effective anticoagulation, has also been shown in patients with antiphospholipid syndrome when taking warfarin.^25^ However, the mechanism of inhibition of thrombin generation differs for the two agents: warfarin reduces functional levels of vitamin-K-dependent coagulation factors, whereas rivaroxaban directly inhibits factor Xa through specific binding to its active site.^28^, ^29^ Warfarin, therefore, affects all phases of thrombin generation equally, whereas rivaroxaban mainly affects the initiation and propagation of thrombin generation, leading to a delay in formation of the prothrombinase complex.^30^ As a result, the thrombin generation curve becomes protracted, which in turn lengthens the lag time and time to peak thrombin generation,^23^, ^30^ and leads to greater ETP than would be expected for the degree of anticoagulation.^23^

The differential effects of warfarin and rivaroxaban were reflected in the treatment effects in this study. On average, in patients who switched from warfarin to rivaroxaban, ETP increased by 100% and time to peak thrombin generation by 70%, whereas peak thrombin generation decreased by 40%. The higher ETP associated with rivaroxaban can be explained by altered reaction kinetics that do not affect thrombotic risk. This conclusion reflects the clinical findings in the phase 3 randomised controlled trials of direct oral anticoagulants,^6^ which are likely to have included patients with antiphospholipid syndrome.^5^

The findings for ETP and peak thrombin generation in RAPS patients at day 42 can be attributed to anticoagulation rather than antiphospholipid antibodies. Indeed, in vitro, the effects of antiphospholipid antibodies on thrombin generation are limited to prolongation of lag time and time to peak thrombin generation.^31^ Our exploratory post-hoc analysis showed no significant interactions between the effects of rivaroxaban and lupus anticoagulant positivity on thrombin generation. Antiphospholipid antibodies might interfere with the anticoagulant action of direct oral anticoagulants, but we have shown no effect with rivaroxaban in in-vitro studies.^31^

A limitation of RAPS is that it was not designed to confirm clinical efficacy and long-term safety. Rather, the trial was designed with a laboratory surrogate outcome measure to assess the mechanism of action of the interventions in these patients. A trial with a primary endpoint of recurrent thrombosis would require a sample of several thousand patients, which is unfeasible for patients with thrombotic antiphospholipid syndrome, and a much longer follow-up period. There was an intended selection bias because we excluded patients who had had venous thromboembolism and developed recurrent events while taking standard-intensity anticoagulation (ie, needing higher-intensity anticoagulation) and those with arterial events. Thus, our cohort seemed to have antiphospholipid antibodies that caused clinical disease at the less aggressive end of the range seen in patients with thrombotic antiphospholipid syndrome. Nevertheless, limiting the selection of patients to those with thrombotic antiphospholipid syndrome and previous venous thromboembolism leading to treatment with standard intensity warfarin ensured a clinically homogeneous study population which is in contrast to two previous, slightly smaller, randomised controlled trials.^32^, ^33^ This feature is an important strength of RAPS. Thrombotic antiphospholipid syndrome is clinically heterogeneous, with the risk of recurrent thrombosis and intensity of anticoagulation being dependent on the clinical phenotype.^2^ Thus, trials, such as RAPS, that involve clinically homogeneous thrombotic antiphospholipid syndrome populations, maximise clinical applicability for subgroups of patients. We caution, therefore, that our results are not applicable to patients with antiphospholipid syndrome and venous thromboembolism who need greater than standard-intensity anticoagulation or with stroke or other arterial thrombosis.

Direct oral anticoagulants have several advantages compared with warfarin. They avoid the need for routine anticoagulation monitoring, which is particularly relevant to antiphospholipid syndrome patients because thromboplastins have variable sensitivity to lupus anticoagulants and, therefore, the INR might not accurately reflect anticoagulation intensity.^22^ If INR instability develops, frequent anticoagulant monitoring will be needed with unpredictable time intervals, and the risk of thrombosis or bleeding will be increased due, respectively, to undercoagulation or overcoagulation. The percentage of time in the therapeutic range for patients in the RAPS warfarin group was only 55% up to day 180. This finding highlights that the predictable anticoagulant effect of rivaroxaban offers a potential advantage in antiphospholipid syndrome patients, but efficacy is dependent upon adherence to the treatment regimen. Unlike treatment with warfarin, where anticoagulation is constant, rivaroxaban leads to peaks and troughs. No range of therapeutic rivaroxaban concentrations have been defined for clinical use. Population pharmacokinetics indicate that peak rivaroxaban concentrations are in the range 160–360 μg/L.^34^ 29 (51%) of 57 RAPS patients had peak therapeutic concentrations of at least 160 μg/L, and three of these had concentrations greater than 360 μg/L. Six (11%) patients had peak concentrations lower than 50 μg/L and were possibly non-adherent.

The absence of new thrombotic events during 6 months of treatment in RAPS justifies our selection of this subgroup of patients with antiphospholipid syndrome and puts into context anecdotal case reports and findings in small case series of recurrent thrombosis after switching patients from warfarin to a direct oral anticoagulant. Of note, 28% of patients in RAPS had triple positivity for lupus anticoagulant and antibodies against cardiolipin and β_2_GPI at baseline and, therefore, had a particularly high-risk antibody profile.^35^

Our findings suggest that in patients with antiphospholipid syndrome who have had previous venous thromboembolism and need standard-intensity anticoagulation (ie, target INR 2·5) the overall thrombotic risk, based on the overall thrombogram, in-vivo coagulation activation markers and clinical outcomes, is not increased with rivaroxaban compared with that related to warfarin. The absence of new thrombosis or major bleeding and low rate of clinically relevant bleeding supports this conclusion. Further studies are required to define the role of direct oral anticoagulants in antiphospholipid syndrome patients, including those with venous thromboembolism who need higher-intensity anticoagulation (ie, those without recurrent venous thromboembolism while taking standard-intensity anticoagulation) or antiphospholipid syndrome patients with stroke or other arterial thrombosis. Overall, rivaroxaban seems efficacious and safe, and might offer a convenient alternative to warfarin in this subgroup of patients with antiphospholipid syndrome.

## Figures and Tables

**Table 1:** Baseline characteristics of trial participants

			**Rivaroxaban (n=57)**	**Warfarin (n=59)**
Demographics				
	Mean (SD) age (years)		47 (17)	50 (14)
	Women		42 (74%)	42 (71%)
	Men		15 (26%)	17 (29%)
	Mean (SD) BMI (kg/m^2^)		28 (6)	30 (6)
Stratification variables				
	SLE		11 (19%)	11 (19%)
	Sites			
		University College London Hospital	23 (40%)	25 (42%)
		Guy's and St Thomas' Hospitals	34 (60%)	34 (58%)
Rivaroxaban dose				
	20 mg once daily		55 (96%)	N/A
	15 mg once daily*		2 (4%)	N/A
Laboratory data				
	Haemoglobin (g/L)		130 (126–135)	137 (134–140)
	Platelet count (× 10^9^/L)		222 (205–240)	220 (204–237)
	International normalised ratio		2·8 (2·6–2·9)	2·7 (2·5–3·0)
	Creatinine clearance (mL/min)		92 (85–100)	95 (88–104)
	Alanine aminotransferase (IU/L)		21 (19–24)	20 (17–22)
Thrombin generation				
	ETP (nmol/L per min)†		555 (497–619)	542 (469–626)
	Lag time (min)		7·3 (6·4–8·2)	7·6 (6·6–8·7)
	Time to peak thrombin generation (min)		10·8 (9·7–12·0)	11·7 (10·3–13·2)
	Peak thrombin generation (nmol/L)		93·8 (78·8–111·7)	79·9 (64·9–98·2)
In-vivo coagulation activation markers				
	Prothrombin fragment 1·2 (pmol/L)		43·3 (38·0–49·3)	43·1 (37·5–49·6)
	Thrombin–antithrombin complex (μg/L)		2·9 (2·5–3·4)	2·7 (2·6–2·9)
	Median (IQR) D-dimer (mg/L FEU)		0·19 (0·19–0·25)	0·19 (0·19–0·22)
Raised in-vivo coagulation activation markers (n)				
	Prothrombin fragment 1·2		0	1
	Thrombin–antithrombin complex		2	2
	D-dimer		3	4
	Any marker		5	6
Thrombotic event with no or subtherapeutic anticoagulation‡				
	Deep vein thrombosis§		32 (56%)	37 (63%)
	Pulmonary embolism		25 (44%)	22 (37%)
Previous bleeding events while taking anticoagulation				
	Major		0	0
	Clinically relevant		0	4 (7%)
aPL (Miyakis categories¶)				
	I (excluding triple-positive aPL)		16 (28%)	19 (32%)
	I (including triple-positive aPL‖)		7 (12%)	12 (20%)
	IIa		30 (53%)	23 (39%)
	IIb		3 (5%)	1 (2%)
	IIc		1 (2%)	4 (7%)
Mean (SD) percentage of time in therapeutic range while taking warfarin**			64 (28)	53 (24)
Mean (SD) ED-5Q-5L quality of life scores				
	Health utility		0·83 (0·21)	0·79 (0·24)
	Health state: VAS††		81 (16)	75 (20)

Data are number (%) or geometric mean (95% CI) unless stated otherwise. ETP=endogenous thrombin potential. SLE=systemic lupus erythematosus. N/A=not applicable. aPL=antiphospholipid antibodies. FEU=fibrinogen equivalent units. ED-EQ-5L=five-level EuroQol-5D. VAS=visual analogue score.

* Given only to patients with creatinine clearance 30–49 mL/min.

† Less than lower limit of detection in three rivaroxaban patients and one warfarin patient.

‡ Recurrent in eight patients assigned to rivaroxaban and nine assigned to warfarin.

§ Rivaroxaban group: lower limb n=23, cerebral venous sinus n=3, subclavian and axillary vein n=1, portal vein n=1, right ventricle n=1, superior vena cava n=1, and retinal vein n=2; warfarin group: lower limb n=27, cerebral venous sinus n=6, axillary vein n=1, portal vein n=1, and retinal vein n=2. Data not collected on whether provoked or unprovoked.

¶ Category I, presence of more than one aPL in any combination; category IIa, presence of lupus anticoagulant alone; category IIb, presence of antibodies against cardiolipin alone; category IIc, presence of antibodies against β_2_ glycoprotein I alone.

‖ 14 rivaroxaban patients, 19 warfarin patients; all patients tested for triple positivity at baseline, thus numbers are higher than for antiphospolipid syndrome-defining aPL; before trial entry, persistence of aPL was established in all patients but triple positivity was not.

** Only calculated if ≥3 international normalised ratio values available; two rivaroxaban patients and seven warfarin patients excluded.

†† One missing value in warfarin group.

**Table 2:** Results from regression models of thrombin generation parameters, in-vivo coagulation activation markers, and quality of life, adherence, and clinical and safety measures in all patients assigned treatment*

			**Rivaroxaban (n=57)**	**Warfarin (n=58)**	**Treatment effect**†**(95% CI)**	**p value**
Thrombin generation at day 42						
	ETP (nmol/L per min)		1086 (957 to 1233)	548 (484 to 621)	2·0 (1·7 to 2·4)	<0·0001
	Lag time (min)		8·9 (8·1 to 9·8)	7·3 (6·7 to 8·0)	1·2 (1·1 to 1·4)	0·0052
	Time to peak thrombin generation (min)		19·2 (17·7 to 20·9)	11·2 (10·3 to 12·1)	1·7 (1·5 to 1·9)	<0·0001
	Peak thrombin generation (nmol/L)		55·6 (46·8 to 66·1)	85·7 (72·3 to 101·5)	0·6 (0·5 to 0·8)	0·00061
In-vivo coagulation activation markers at day 42						
	Prothrombin fragment 1.2 (pmol/L)		93·6 (82·1 to 106·9)	45·6 (40·1 to 52·0)	2·1 (1·7 to 2·5)	<0·0001
	Thrombin–antithrombin complex (μg/L)		2·4 (2·3 to 2·6)	2·6 (2·5 to 2·8)	0·9 (0·9 to 1·0)	0·14
	D-dimer (mg/L fibrinogen equivalent units)		0·19 (0·19 to 0·23)	0·19 (0·19 to 0·20)	0 (0 to 0)	1
	Raised concentrations (also raised at baseline [n])					
		Prothrombin fragment 1.2 (pmol/L)	2 (0)	0	N/A	N/A
		Thrombin–antithrombin complex (μg/L)	0	3 (1)	N/A	N/A
		D-dimer (mg/L FEU)	2 (1)	4 (1)	N/A	N/A
		Any marker	3 (1)	6 (2)	N/A	N/A
Adherence at day 42						
	Median (IQR) rivaroxaban (μg/L)		162 (101 to 245)	N/A	N/A	N/A
	Factor X amidolytic (IU/dL)		N/A	25·3 (23·5 to 27·3)	N/A	N/A
	International normalised ratio		N/A	2·7 (2·6 to 2·9)	N/A	N/A
Mean (SD) time in therapeutic range at day 180 (%)‡			N/A	55 (23)	N/A	N/A
Mean (SE) ED-5Q-5L quality of life scores at day 180						
	Health utility		0·82 (0·02)	0·78 (0·02)	0·04 (−0·02 to 0·09)	0·19
	Health state: VAS		80 (1·8)	73 (1·8)	6·5 (1·4 to 11·5)	0·013
New thrombotic events at day 210						
	Deep vein thrombosis		0	0	N/A	N/A
	Pulmonary embolism		0	0	N/A	N/A
	Arterial thrombosis		0	0	N/A	N/A
	Other		0	0	N/A	N/A
	Any combination		0	0	N/A	N/A
Bleeding events at day 210 ¶						
	Major		0	0	N/A	N/A
	Clinically relevant		3 (5%)	2/55 (4%)	1·7 (−5·9 to 9·3)	N/A
	Minor		10 (18%)	8/55 (15%)	3·0 (−10·5 to 16·5)	N/A
	Unclassified, insufficient information		1 (2%)	0	1·8 (−1·7 to 5·3)	N/A
	Site of bleed§					
		Intracranial	1‖	0	N/A	N/A
		Skin (bruise)	3	0	N/A	N/A
		Oral	0	1	N/A	N/A
		Nasal	5	3	N/A	N/A
		Vaginal	1	0	N/A	N/A
		Rectal	0	3	N/A	N/A
		Lower ureteric	1	0	N/A	N/A
		Other	9	7	N/A	N/A
Adverse events at day 210¶						
	SAE**		4 (7%)	3/55 (5%)	1·5 (−7·5 to 10·5)	N/A
	SAR		0	1/55 (2%)	−1·8 (−5·3 to 1·7)	N/A
	SUSAR		0	0	N/A	N/A

Data for the treatment groups are number (%) or geometric mean (95% CI) unless stated otherwise. ETP=endogenous thrombin potential. FEU=fibrinogen equivalent units. N/A=not applicable. ED-EQ-5L=five-level EuroQol-5D. VAS=visual analogue score. SAE=serious adverse events. SAR=serious adverse reactions. SUSAR=suspected unexpected serious adverse reaction.

* Except for one patient who withdrew before day 42 in the warfarin group.

† Estimated as ratio of rivaroxaban to warfarin for thrombin generation and as the difference between treatments (rivaroxaban–warfarin) for other outcomes. Regression models are adjusted for stratification variables and baseline values of each variable.

‡ Includes only patients with at least three international normalised ratio measurements.

§ Includes patients with bleeding episodes at more than one site; only most severe reported here.

¶ Four patients (two withdrawals, one lost to follow-up, and one death) in the warfarin group did not reach day 210.

‖ Not judged to be related to treatment; the event pre-dated the trial.

** SAE grades are described in the main text.
