# Supplementary material for: Rivaroxaban versus warfarin to treat patients with thrombotic antiphospholipid syndrome, with or without systemic lupus erythematosus (RAPS): a randomised, controlled, open-label, phase 2/3, non-inferiority trial
Source: Lancet Haematol. 2016 Aug 25;3(9):e426–36. doi: 10.1016/S2352-3026(16)30079-5 (PMC5010562; doi:10.1016/S2352-3026(16)30079-5)
Supplement: Supplementary appendix [file mmc1.pdf]

LANCET Haematology

Supplementary appendix

This appendix formed part of the original submission and has been peer reviewed. We post it as supplied by the authors.

Supplement to: Cohen H, Hunt BJ, Efthymiou M, et al, for the RAPS trial investigators. Rivaroxaban versus warfarin to treat patients with thrombotic antiphospholipid syndrome, with or without systemic lupus erythematosus (RAPS): a randomised, controlled, open-label, phase 2/3, non-inferiority trial. *Lancet Haematol* 2016; **3:** e426–36.

Supplementary Appendix

Rivaroxaban versus warfarin to treat patients with thrombotic antiphospholipid syndrome, with or without systemic lupus erythematosus (RAPS): a randomised, controlled, open-label, phase 2/3, non-inferiority trial

H Cohen et al 2016

Contents **Page**

A. Glossary……………………………………………………………………………………………...3

B. Trial Steering Committee members…………………………………………………………..……......5

C. Independent Data Monitoring Committee members…………………………….……….…………………5

D. RAPS laboratory criteria for diagnosis of antiphospholipid antibodies: additional information……………..6

E. Regimen for patients converting from warfarin to rivaroxaban: additional information ……………………8

F. Blinded review of bleeding events…………………………………………………………………………10

G. Statistical Analysis Plan…………………………………………………………………………………11

H. RAPS exploratory analyses……………………………………………………………………………...22

Table S1: LA summary

Table S2: Results of regression models adjusted by stratification variables, baseline values, LA and treatment-by-LA interaction.

I. Transforming Data………………………………………………………………………………………….24

J. Withdrawals, losses to follow-up, and missing outcome data……………………………………………...25

Table S3: Summary of participants and reasons for not attending at each time-point

Table S4: Proportion of cases excluded from the analyses by outcome measure and treatment arm

K. Treatment exposure………….………………………………………………………………………......28

Table S5: Measures of treatment exposure by treatment allocated and assessment time-point

L. RAPS Trial Investigators..………………………………………………………………………………….30

M. Research staff who assisted with patient recruitment, data collection, and data management………………..31

N. References…………………………………………………………………………..……………….......33

A. Glossary

Adequate contraception

Adequate contraception is defined as hormonal contraception or barrier method contraception.

Subtherapeutic anticoagulant therapy

Patients who develop a new venous thromboembolic event while on warfarin with an INR (International Normalised ratio) of <2.0 (target INR 2.5, i.e. range 2.0-3.0) will be considered to have had the event when sub-therapeutically anticoagulated. The same would apply if the patient was on a dose of another anticoagulant (e.g. low molecular weight heparin) administered at less than the manufacturer's recommended therapeutic dose for VTE at the time of the event.

Thrombotic APS

Thrombotic APS is defined as venous thromboembolism (VTE) associated with persistent positivity of one or more antiphospholipid antibodies (aPL; i.e. lupus anticoagulant, IgG and/or IgM anticardiolipin and/or anti beta 2 glycoprotein 1 antibodies > the 99th centile.

Persistent aPL positivity is defined as aPL present on at least two consecutive occasions at least 12 weeks apart as defined in the International (Sydney) Consensus Statement criteria.

Clinical outcome events

All thrombotic events, defined below, will be classified by the PI at the trial site.

Venous thromboembolism

The diagnosis of symptomatic recurrent venous thromboembolism (VTE), i.e. deep-vein thrombosis (DVT) or non-fatal or fatal pulmonary embolism (PE) is based on the following:
DVT is diagnosed objectively on venous doppler or duplex scanning or venography.
PE is diagnosed objectively on computed tomography pulmonary angiogram (CTPA) or ventilation/perfusion (V/Q) lung scanning.
Fatal PE: the diagnosis of fatal PE is based on objective diagnostic testing, autopsy, or death which cannot be attributed to a documented cause and for which PE cannot be ruled out (unexplained death).

Other thrombotic events

Arterial thrombosis/thromboembolism

Stroke is defined as a sudden focal neurologic deficit of presumed cerebrovascular aetiology that persists beyond 24 hours and is not due to another identifiable cause. An event matching this definition but lasting less than 24 hours is considered to be a transient ischemic attack. The diagnosis of stroke is based on brain imaging (computed tomography or magnetic resonance imaging).
Systemic embolism is defined as abrupt vascular insufficiency associated with clinical or radiological evidence of arterial occlusion in the absence of another likely mechanism (e.g. atherosclerosis, instrumentation, or trauma).

Myocardial infarction (MI) is defined by typical symptoms, cardiac biomarker elevation and electrocardiogram changes, or confirmation at autopsy.
Deaths are classified as either vascular (e.g. due to stroke, embolism, myocardial infarction, or arrhythmia) or non-vascular (e.g. malignancy or infection).

Microvascular thrombosis
Thrombosis in the microvasculature, generally associated with evidence of organ dysfunction in patients with thrombotic APS, is diagnosed on histological examination of a tissue biopsy.

Lack of Drug Effect (LODE)
Non-serious adverse events (AE) will be considered due to LODE if the investigator explicitly states that the non-serious adverse event occurred due to the drug not working. A non-serious AE would not be considered to be due to LODE if e.g. there has been a medication error (insufficient dose was used, prescribed dosing regimen was not followed), handling error (instructions for use not followed, product stored incorrectly or used after expiry date) or there was a misuse or abuse of the product.

B. RAPS Trial Steering Committee (TSC) membership

Independent members:

Professor Michael Greaves: Chair, Head of School of Medicine, University of Aberdeen, Foresterhill, Aberdeen, AB25 2ZD, UK

Dr Christopher J Edwards: Expert member, Consultant Rheumatologist and Honorary Senior Lecturer, Associate Director NIHR Wellcome Trust, University Hospital Southampton NHS Foundation Trust, Mailpoint 218, Southampton General Hospital, Tremona Road, Southampton, SO16 6YD, UK

Kate Hindle: Lay member, Manager, Hughes Syndrome Foundation, Conybeare House, Guy's Hospital, London, SE1 9RT, UK

Non-independent members:

Dr Hannah Cohen, RAPS Chief Investigator

Professor Caroline Doré, RAPS Lead Trial Statistician

C. RAPS Independent Data Monitoring Committee (IDMC) Membership

Independent members:

Professor Peter Maddison: Chair, Professor of Musculoskeletal Medicine, School of Medical Sciences, Bangor University, North West Wales NHS Trust, Brigantia Building, Penrallt Road, Bangor, LL57 2AS, UK

Dr Will Lester, Consultant Haematologist, Queen Elizabeth and Birmingham Women's Hospital and Honorary Senior Lecturer, School of Clinical and Experimental Medicine, Vincent Drive, University of Birmingham, Birmingham B15 2TT, UK

Dr Steve Kitchen, Scientific Director of the UK NEQAS for Blood Coagulation (from 25 April 2014), 3rd Floor Pegasus House, 463A Glossop Road, Sheffield S10 2QD, UK

Dr Martyn Lewis, Reader in Biostatistics, Arthritis Research UK Primary Care Centre, Primary Care Sciences, Keele University, Staffordshire, ST5 5BG, UK

Non-independent statisticians:

Professor Caroline Doré, RAPS Lead Trial Statistician

Yvonne Sylvestre, RAPS Trial Statistician

D. RAPS laboratory criteria for antiphospholipid antibodies (aPL): additional information

Persistent antiphospholipid antibodies

i.e. present on two or more occasions at least 12 weeks apart

The diagnosis of aPL is based on the Sapporo (Sydney) International Consensus Criteria, and International Society of Thrombosis and Haemostasis Scientific Subcommittee (ISTH SCC) and British Committee for Standards in Haematology (BCSH) guidance.1-3

Lupus anticoagulant (LA)

Patients not receiving anticoagulants (warfarin/other vitamin K antagonist (VKA) or therapeutic dose heparin/low molecular weight heparin (LMWH))

Testing should be performed using accepted testing such as the dilute Russell's viper venom time (DRVVT), the dilute activated partial thromboplastin time (APTT) assay or other appropriate assays as per BCSH/ISTH guidelines. Mixing tests and confirmatory procedures (high phospholipid, different phospholipid composition, or platelet neutralisation procedure) must be included to demonstrate an inhibitor (mixing tests) and show phospholipid dependence.

Lupus anticoagulant (LA) testing in patients on VKA

At least one of the following should be positive:

1) Taipan snake venom time (TVT) with a platelet neutralisation procedure or ecarin time as confirmation.

2) DRVVT: Screening and confirmatory steps on equal volume (50:50) mixtures of patient and normal plasma. If the screening step on the mixture is abnormal, this may be taken as grounds for considering that an inhibitor is present and the confirmatory step will demonstrate phospholipid dependence. The calculation of results should be according to the laboratory standard operating procedure (SOP)/manufacturers' recommendations.

Points to note:

- The DRVVT and TVT are appropriate, but other methods may also be used (e.g. Acticlot dilute PT).
- Due to the dilution effect, a negative result in mixing studies does not exclude the presence of a LA
- Patients on low molecular weight/unfractionated heparin may have false positive results, therefore do not include these
- The APTT is not suitable due to the prolongation caused by warfarin
- The DRVVT may be performed on unmixed plasma if the INR is <1.5, and should be performed on a 50:50 mix with normal plasma if the INR is 1.5-3.0.

Anticardiolipin (aCL) antibodies

IgG and/or IgM

Moderate/high positive: > the 99th centile

Anti-β2 glycoprotein I (aβ2GPI) antibodies

IgG and/or IgM

Moderate/high positive: > the 99th centile

E. Regimen for patients converting from warfarin to rivaroxaban: additional information (explanatory notes added in bold font)

Regimen for patients converting from warfarin to rivaroxaban

- For patients randomised to rivaroxaban, the warfarin should be stopped on the day of conversion from warfarin to rivaroxaban
- If the INR is ≤2.5, the rivaroxaban should be started the following morning
- If the INR is 2.6 to ≤3, the rivaroxaban should be started 2 days later in the morning
- If the INR is >3, the INR should be rechecked as required and the rivaroxaban should be started, as a morning dose:
  - when the INR is 2.6 to ≤3, the rivaroxaban should be started 2 days later in the morning
  - when the INR is ≤2.5, the rivaroxaban should be started the following morning

Additional information (explanatory notes added in bold font)

1. The protocol is entirely in accordance with the summary of product characteristics (SPC) and should be used in that context.

The SPC for Xarelto (rivaroxaban) states:

"For patients treated for DVT, PE and prevention of recurrence, VKA treatment should be stopped and Xarelto therapy should be initiated once the INR is ≤ 2.5."

2. The protocol states:

"Regimen for patients converting from warfarin to rivaroxaban:

- For patients randomised to rivaroxaban, the warfarin should be stopped on the day of conversion from warfarin to rivaroxaban. **This means that patients randomised to rivaroxaban should not take the next due dose of warfarin after randomisation, which will generally be either that evening or the following morning.**
- If the INR is ≤2.5, the rivaroxaban should be started the following morning. **This means that if the INR is ≤2.5 on the baseline sample taken on the day of randomisation, then the rivaroxaban should be started the next morning (the patient having not taken the warfarin the evening of randomisation/the following morning - and of course from then onwards). Therefore the rivaroxaban will be started around 24 hours or more after the last warfarin dose.**
- If the INR is 2.6 to ≤3, the rivaroxaban should be started 2 days later in the morning. **This means that if the INR is 2.6 to ≤3, the rivaroxaban will be started around 48 hours or more after the last dose.**

- If the INR is >3, the INR should be rechecked as required and the rivaroxaban should be started, as a morning dose:
  - when the INR is 2.6 to ≤3, the rivaroxaban should be started 2 days later in the morning
  - when the INR is ≤2.5, the rivaroxaban should be started the following morning

NB If the INR is between 2.5 and 2.6, then transcription of the INR result should be corrected upwards to 2.6 if the result is 2.55 and above and corrected downwards to 2.5 if the result is between 2.51-2.54.

Note: When converting a patient from warfarin to rivaroxaban, the INR may not be representative of anticoagulant effect after rivaroxaban dosing. Therefore, the INR should not be used as a measure of anticoagulant activity of rivaroxaban.

F. Blinded review of bleeding events

Bleeding events across all sites were psuedoanonymised and reviewed by a single investigator, removing the potential for bias and inter-operator variation. Bleeding events were categorised as 'minor,' 'clinically relevant non-major,' and 'major' using the following criteria as defined in the RAPS Protocol Glossary (p 7-8). There was no major bleeding, so the bleeds were categorised as minor or clinically relevant non-major.

Major bleeding

Major bleeding is defined as clinically overt bleeding associated with any of the following: fatal outcome, involvement of a critical anatomic site (intracranial, spinal, ocular, pericardial, articular, retroperitoneal, or intramuscular with compartment syndrome), fall in hemoglobin concentration of at least 20 g/L, transfusion of 2 or more units of red blood cells, or permanent disability.

Clinically relevant non-major bleeding

Clinically relevant non-major bleeding is defined as overt bleeding not meeting the criteria for major bleeding but associated with medical intervention, unscheduled contact (visit or telephone) with a physician, temporary interruption of study drug (i.e. delayed dosing), or associated with any other discomfort such as pain or impairment of activities of daily life.

Minor bleeding

Bleeding events that do not meet the criteria of major or clinically relevant non-major bleeding are defined as minor.

G. Statistical Analysis Plan; version 1.0, 24 Feb 2015

1 ABBREVIATIONS

Acronyms	Meaning
APS	Antiphospholipid Syndrome
CRF	Case Report Form
CI	Confidence Interval
CIVC	Crosswalk Index Value Calculator
ETP	Endogenous Thrombin Potential
INR	International Normalised Ratio
ITT	Intention-to-treat
MAR	Missing at Random
MCAR	Missing Completely at Random
OD	Once Daily
RHU	Research Haemostasis Unit
SAE	Serious Adverse Event
SAP	Statistical Analysis Plan
SLE	Systemic Lupus Erythematosus
eSMF	Statistical Master file stored electronically
TMF	Trial Master File
TG	Thrombin Generation
TTR	Percentage Time in Therapeutic Range
VTE	Venous Thromboembolism

2 ABSTRACT – BACKGROUND AND DESIGN

Aim and objectives: The aim of this study is to compare the efficacy and safety of a fixed-dose anticoagulant drug (rivaroxaban) with that of warfarin in patients with thrombotic antiphospholipid syndrome (APS), with or without systemic lupus erythematosus (SLE).

The primary objective is to demonstrate that the intensity of anticoagulation achieved with rivaroxaban is not inferior to that with warfarin, by measurement of the dynamics of *ex vivo* thrombin generation using the thrombin generation test (TG) with the endogenous thrombin potential (ETP) as the key parameter.

Population studied: 116 patients with thrombotic APS, with or without SLE.

Trial design: RAPS is a two arm unblinded phase II/III prospective randomised controlled non-inferiority clinical trial. Patients on warfarin will be randomly allocated to either continue with their standard care or to change to rivaroxaban. Each patient will have a six month treatment period and a final visit 30 days after the end of trial treatment.

Sample size: Using a one-sided 2.5% significance level and 80% power it is calculated that 51 subjects per group are required for the study. Allowing for a 12% drop-out rate, a total of 116 patients will need to be randomised.

Randomisation: Random permuted blocks of varying length will be used to equally allocate participants to either remain on warfarin or switch to rivaroxaban, stratified by site and patient type.

This trial will not be blinded or masked as potentially this could be unsafe for the participants.

3 OUTCOME MEASURES

3.1 Primary outcome

The primary outcome measure is the percentage change in ETP from randomisation to day 42. ETP is measured in nmol/L per minute (nM/min).

3.2 Secondary outcomes

a) Efficacy outcomes

1. Frequency of patients experiencing recurrent venous thromboembolism (VTE) alone up to day 180
2. Frequency of patients experiencing a composite of recurrent VTE and other thrombotic events up to day 180
3. Percentage change from randomisation to day 42 of the parameters derived from the thrombin generation curve: lag-time, time to peak, peak thrombin concentration.

4. Percentage change from randomisation to day 42 of markers of *in vivo* coagulation activation: prothrombin fragment 1.2, thrombin-antithrombin complex and D-dimer.

b) Safety outcomes

1. Frequency of Serious Adverse Events (SAEs) reported up to day 210.
2. Frequency of patients experiencing bleeding up to day 210. Bleeding will be categorised as: major, clinically relevant non-major, minor.

c) Quality of life as measured by the EQ-5D-5L questionnaire at day 180.

d) Laboratory measures of compliance

1. A rivaroxaban anti-Xa assay, in patients on rivaroxaban at Day 42
2. An International Normalised Ratio (INR) and factor X amidolytic assay, in patients on warfarin at baseline and at Day 42.
3. Percentage time in therapeutic range (TTR) between baseline and day 180 in patients on warfarin.

3.3 Scoring outcome measures

3.3.1 EQ-5D-5L

The EQ-5D-5L consists of a self-reported matrix comprising 5 items or dimensions (i.e., mobility, self-care, usual activities, pain-discomfort and anxiety-depression) rated on 5-point scales ranging from 0 to 4 and a self-rated health state 100mm visual analogue scale (VAS).[4] Respondents' ratings can be combined into a single health utility score.

Scoring: Value sets based on preferences directly elicited from representative general population samples to derive the EQ 5D 5L health utility score are not yet available. If they are not disseminated by the time follow-up CRF forms are completed, we shall use the UK value sets of the "EQ-5D-5L Crosswalk Index Value Calculator (CIVC),[5] available for download at the EuroQol Group website, to obtain the EQ 5D 5L health utility score. As its name implies, the CIVC is a "crosswalk" between the EQ-5D-3L value sets and the new EQ-5D-5L descriptive system resulting in crosswalk value sets for the EQ-5D-5L.

Missing items: If the instrument has no more than one item missing (i.e. ≤20%), then we shall impute the missing item by the weighted mean of the completed items and score it as described above. If the instrument has more than 1 missing item (i.e.>20%), then the score will be set to missing.

3.3.2 TTR

The time in therapeutic range (TTR) is a method of summarising INR control over time.

Scoring: We shall use the Rosendaal Method[6] for computing the percentage time in therapeutic range. In this INR-specific method, person-time is calculated by incorporating the frequency of INR measurements and their actual values and assuming that changes between consecutive INR measurements are linear over time.

The TTR (%) will be estimated using the formula:

$$TTR(\%) = \frac{\sum_{i=2}^{n} \left(\frac{TSwR}{TS}\right) \times D}{\sum_{i=2}^{n} D} \times 100 \qquad for\ i = 2, \ldots.n$$

Where:

TS = Amount of the total shift between INR measurements

TSwR = Amount of the total shift between INR measurements that is within the therapeutic range (i.e., 2.0 – 3.0)

D = Number of days since last visit

i = Visit number

4 DATA

4.1 CRF and variables

Full details of data collection and timing are described in the trial protocol (5.0 dated 02 September 2014). Copies of CRFs are included in the Trial Master File (TMF).

4.2 Management of datasets

At the time of analysis:

- A copy of each dataset will be prepared by the Trial Statistician (frozen dataset, downloaded from the database) and saved in section 3 'Annotated output from statistical analysis programme' of the Statistical Master File (eSMF).
- If necessary, data can be added to or amended in the MACRO database after data download.

- If any outstanding queries are resolved during the analysis that relate to data in the frozen dataset (e.g., problems that are found during analysis or amended CRFs that are returned to the University College London Comprehensive Clinical Trials Unit (CCTU), the MACRO database and frozen dataset should both be updated.
- If any outstanding data queries are resolved while the analysis files are being prepared (when only a practice dataset has so far been copied), the changes need only be made to the MACRO database and an updated frozen copy created in section 3 of the eSMF.

4.3 Data completion and schedule

The last patient for the RAPS trial was randomised on the 11th November 2014. All forms for 210-day follow-up should therefore be available by mid June 2015, with leeway of 14 days for study visits.

4.4 Data verification

Data verification, consistency and range checks are performed during data entry, as well as checks for missing data (copies of these checks can be found in the TMF). Additional range, consistency and missing data checks will be performed when the datasets for analysis are constructed, before the statistical analysis is performed. All variables will be examined for unusual, outlying, unlabelled or inconsistent values.

Any problems with trial data will be queried with the Trial Manager. If possible, data queries will be resolved; although it is accepted that due to administrative reasons and data availability a small number of problems will continue to exist. These will be minimised.

4.5 Data coding

Details of the variables, including variable coding lists are included in the metadata which forms part of the TMF.

5 SAMPLE SIZE ESTIMATION

5.1 Type of Comparison and Hypothesis

RAPS is designed as a non-inferiority trial, to demonstrate that the intensity of anticoagulation in patients on Rivaroxaban is not inferior to that obtained with warfarin as assessed by the ETP. Rivaroxaban would be regarded as non-inferior to warfarin if the percentage change in ETP is not more than 20% higher (i.e. less anticoagulant effect) than that for warfarin. The non-inferiority limit of 20% is based on the inter site assay variability of test performance[7] and on clinical relevance.

The null hypothesis (H_0) is that Rivaroxaban is inferior to warfarin with respect to the mean percentage change in ETP at six weeks:

H_0: $|\mu_R - \mu_W| \geq 20\%$

The alternative hypothesis (H_1) is that Rivaroxaban is not inferior to warfarin with respect to the mean percentage change in ETP at six weeks:

H_1: $|\mu_R - \mu_W| < 20\%$

Where: μ_R = mean percentage change in ETP in the rivaroxaban group

μ_W = mean percentage change in ETP in the warfarin group

In order to conclude non-inferiority, we need to reject the null hypothesis.

5.2 Sample size: Primary outcome

Using information on the ETP in patients on warfarin at a comparable INR range the standard deviation of % change in ETP was 36% six weeks after starting warfarin treatment.[8] Using a one-sided 2.5% significance level and 80% power it is calculated that 51 patients per group are required for the study. Based on our experience with the first 84 patients recruited, 2 patients have been lost to follow-up or withdrawn before the first trial visit post randomisation, and 5 patients have not provided paired ETP values. If we conservatively allow for 12% non-evaluable patients, it is anticipated that a total of 116 patients will need to be randomised. The sample size has not been adjusted for non-compliance since, unlike in a superiority trial, it does not necessarily reduce the power for a non-inferiority design.[9]

5.3 Secondary outcomes: efficacy and safety

The trial is not powered to detect differences between the two randomised groups for secondary efficacy (i.e. recurrent thrombosis) or safety endpoints (i.e. SAEs and bleeding events).

6 ANALYSIS PRINCIPLES

6.1 Intention-to-treat (ITT) or per-protocol?

To retain the validity of the randomisation process all analyses will be by intention-to-treat, and will include all consented patients for whom outcomes are available.

In addition, a per-protocol analysis will be performed for the primary outcome (see section 7.4.2)

6.2 Significance level of tests

All confidence intervals will be 95% and two-sided. Statistical hypothesis tests will use a two-sided p value of 0.05, unless otherwise specified. There will be no formal adjustment of p values for any interim analyses performed.

6.3 Baseline comparability

Baseline characteristics will be summarised by randomised group.

6.4 Adjustment for design factors

Since randomisation was stratified by site (i.e. UCLH and Guy's and St. Thomas') and type of patient (i.e. SLE, non-SLE), analyses of continuous outcomes will involve adjustment for these two factors as recommended in ICH E9.[10] Treatment effects are then estimated conditional on site and type of patient. The model for the primary outcome will be adjusted for baseline ETP values. Similar adjustments will be made for other continuous secondary outcomes if a baseline value is available.
Adjustment for design factors will not be made for binary secondary outcomes since there are likely to be too few events to fit logistic regression models.

6.5 Follow-up and losses to follow-up: handling missing data

The sample size estimation assumed 12% of patients would not provide evaluable ETP measurements. We have examined our actual retention rate to the trial and at the time of writing this report two participants have dropped out from the 84 randomised to date. However, the primary outcome is not available for five patients as the TG test failed to give a result. This might be due to the blood samples being haemolysed. Haemolysis occurred in vitro, this means it is caused by the way in which the blood samples are drawn and treated and not related to any medical condition. Hence, patients are not systematically different from those providing viable samples and therefore it is reasonable to assume that these results are missing completely at random (MCAR).

Allowing for a conservative 12% drop out rate, data will be assumed to be MCAR and we will perform a complete case analysis. This approach is consistent with current practice; in a randomised controlled trial setting with missing data only in the outcome measure, multiple imputation offers no advantage over complete case analysis using analysis of covariance (ANCOVA) or mixed models, where the fraction of missing data is small and MAR can reasonably be assumed.[11]

6.6 Summarising models

Where possible, analysis of outcomes will involve a parametric model. Treatment effect estimates will be presented as regression coefficients and 95% confidence intervals.

If necessary, ETP and other continuous outcomes which are not expected to be normally distributed will be log transformed for the statistical analysis. Estimates and 95% confidence intervals on the log scale will be back transformed to percentage changes for presentation as the difference between two logarithms is the log of their ratio.

7 ANALYSIS DETAILS

The results of the analyses will be reported following the principle of the ICH E3 guidelines[12] on the Structure and Content of Clinical Study Reports.

7.1 Recruitment and follow-up patterns

Patients screened but not enrolled in the Trial and reasons for exclusions will be reported by site.

Recruitment will be presented by year and site.

The number of outcomes completed – excluding patients who have been withdrawn from therapy and were unwilling to continue follow-up, will be reported by treatment group.

The number of patients who have been withdrawn from therapy, were unwilling to continue follow-up or died while on study, will be reported by treatment group.

7.2 Baseline Characteristics

Baseline characteristics will be reported for each treatment arm. Summary measures for the baseline characteristics of each group will be presented as mean and standard deviation for continuous (approximate) normally distributed variables, medians and interquartile ranges for non-normally distributed variables, and frequencies and percentages for categorical variables.

7.3 Trial treatment

Adherence to treatment will be summarised by treatment group.

7.4 Analysis methods

7.4.1 Primary analysis

We shall use a linear regression model to estimate the difference in ETP between the two treatments (rivaroxaban - warfarin) at day 42 together with a two-sided 95% confidence interval, adjusting for the stratification variables (i.e. site and patient type). Baseline ETP will be included as a covariate. ETP will be log transformed for the statistical analysis. Results will be back transformed and presented as geometric means (GM) and 95% confidence intervals (CI), or ratios and 95% CI. If the upper end of the 95% CI does not cross the non-inferiority limit of 20%, then rivaroxaban will be regarded as non-inferior (see B and C in the figure below). If the mean ratio is less than one and the 95% CI does not include a ratio of one then we can conclude that rivaroxaban is superior to warfarin (see A in the figure below).

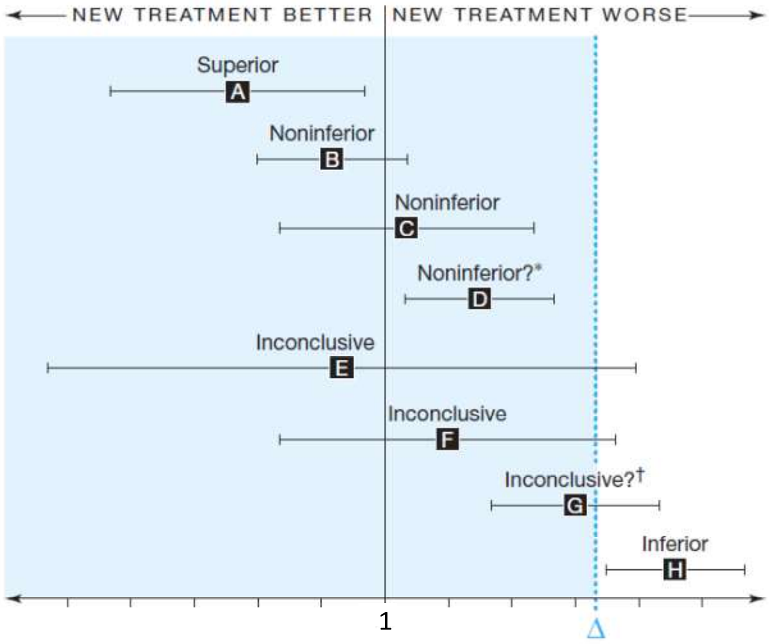

7.4.2 Sensitivity analyses for the primary outcome

A sensitivity analysis will be performed by fitting a tobit regression model which can handle censored values, below the limit of detection of the assay. In addition, a per-protocol analysis will be performed to assess the treatment effect in adherent patients.

7.4.3 Secondary analyses

As stated in section 5 the trial is not powered to detect differences between the two randomised groups for secondary efficacy or safety endpoints. Therefore, for secondary outcomes the differences between the two groups will be summarised using estimates and confidence intervals.

a) Efficacy

Continuous secondary outcome measures of the parameters derived from the thrombin generation curve (i.e. lag-time, time to peak, peak) and markers of *in vivo* coagulation activation (i.e. prothrombin fragment 1.2, thrombin-antithrombin complex and D-dimer) will be analysed using a linear regression model to estimate differences between the two treatments (rivaroxaban - warfarin) at Day 42 together with a two-sided 95% confidence interval. Stratification variables (i.e. site and patient type) and baseline values will be included as covariates in the models. Estimates of treatment effects with 95% CI will be presented.

Differences between the two arms for binary efficacy outcomes 1 and 2 described in section 3.2 will be analysed using Fisher's exact tests. Summary measures will be the number (%) of patients with an event in each group. Treatment effects will be estimated by the difference in event rates and 95% CI for the differences.

b) Safety

Summary measures will be the number (%) of patients with a SAE in each group. Treatment effects will be estimated by the difference in event rates and 95% CI for the differences.

c) Compliance

The rivaroxaban anti-Xa assays in the patients on rivaroxaban (at Day 42) and the INRs and factor X amidolytic assays in patients on warfarin (at baseline and Day 42) will be correlated, using Pearson's correlation coefficient or Spearman's rank correlation coefficient if data is clearly not Normally distributed (following a log transformation, if that improves Normality), with log transformed ETP to assess whether there are any relationships. In addition, ETP and anti-Xa assays in patients on rivaroxaban will be correlated with the time elapsed since rivaroxaban ingestion on the day of blood sample collection.

Time in Therapeutic Range (TTR) for patients on warfarin will be summarised using means and standard deviations.

7.4.4 Regression diagnostics

Diagnostic checks to assess the appropriateness of the regression models fitted will be through the use of residual plots. We will plot:

- Histograms and probability plots to assess normality
- Scatterplots of residuals against fitted values to assess constant variance and linearity, and to identify potential outliers

7.4.5 Subgroup analyses

There are no planned subgroup analyses.

7.5 Quality of Life

Quality of life as measured by the EQ-5D-5L will be analysed using a linear regression model to estimate the differences between the two treatments (rivaroxaban - warfarin) at Day 180. Stratification variables (i.e. site and patient type) and baseline measurements will be included as covariates in the models. Estimates of treatment difference with 95% CI will be presented.

H. RAPS exploratory analyses

Adjusting TG parameters for LA status

We estimated linear regression models to estimate the difference in TG parameters between the two treatments (rivaroxaban - warfarin) at day 42 together with a two-sided 95% confidence interval, adjusting for the stratification variables (i.e. site and patient type) and LA status. Baseline values for each respective outcome were included as covariates in the models.

We also performed a subgroup analysis on LA status (i.e. LA positive vs. not detected) by testing for the interaction between treatment and LA status within each regression model to answer the question of whether any observed differences between treatment groups vary depending on LA status.

As for the primary and secondary analyses, outcomes were log transformed for the exploratory analyses. Results were back transformed and presented as geometric means ratios (GMR) and 95% confidence intervals (CI).

The exploratory subgroup analysis did not demonstrate any significant interactions between the effects of rivaroxaban and LA positivity at baseline for any of the four TG parameters. Results are presented in the tables below.

Table S1: LA summary

Baseline LA	Rivaroxaban	Warfarin
Positive	25 (44%)	31 (53%)
Not detected	32 (56%)	28 (47%)

Table S2: Results of regression models adjusted by stratification variables, baseline values, LA and treatment-by-LA interaction.

			95% CI		
		Effect*	Lower	Upper	p-value
ETP	LA	0.96	0.74	1.24	0.762
	Treatment-by-LA	0.94	0.65	1.36	0.749
Lag	LA	1.10	0.89	1.37	0.376
	Treatment-by-LA	1.05	0.79	1.40	0.720
Time to peak	LA	1.12	0.93	1.35	0.225
	Treatment-by-LA	0.98	0.77	1.25	0.881
Peak	LA	0.90	0.64	1.28	0.559
	Treatment-by-LA	1.02	0.62	1.69	0.933

*Geometric mean ratio (rivaroxaban:warfarin)

I. Transforming data

Primary and continuous secondary efficacy outcomes were log transformed for the statistical analyses to achieve normality. Results were back-transformed by anti-logging at the end of the analysis to be meaningfully interpreted. It is important for the non-statistical audience to understand that when estimating the difference between two means in log-transformed data, the back-transformation produces a ratio. For instance, to estimate the mean difference in ETP between the two treatments (rivaroxaban - warfarin) at day 42 we fitted a linear regression model adjusted for stratification variables (i.e. site and patient type) and baseline values in the log transformed data. The results yield a difference in log means (rivaroxaban - warfarin) of 0·7 (95% CI 0·5 to 0·9, $p < \cdot 0001$). When back-transforming the results by anti-logging the geometric mean ratio (GMR) rivaroxaban: warfarin of the changes in ETP from baseline to day 42 in the two groups was 2·0 (95% CI 1·7 to 2·4, $p < \cdot 0001$) which indicates that the mean % change in ETP from baseline to day 42 significantly doubles in patients on rivaroxaban in comparison to patients treated with warfarin. This can be expressed in terms of the percentage change, we can say that for those patients who switch their treatment from warfarin to rivaroxaban, we can expect to see a 100% (95% CI 70 to 140, $p < \cdot 0001$) significant increase in the mean ETP.

J. Withdrawals, losses to follow-up, and missing outcome data

We randomised 116 patients; 57 to receive rivaroxaban and 59 to continue to receive warfarin.

In the rivaroxaban group:

Patient T014 did not attend (DNA) the day 180 trial visit after experiencing an intestinal perforation.

In the warfarin group:

- Patient T042 withdrew before the first trial visit (day 42) due to travel distance and time related issues, and because she did not get rivaroxaban. No follow-up data was obtained.
- Patient T047 withdrew after the first trial visit (day 42) as the general practitioner (GP) offered to start the patient on rivaroxaban outside the trial. This patient, therefore, did not continue on the allocated treatment. The patient did not attend the day 90 visit, returned to the clinic for the day 180 assessment and did not attend the day 210 visit.
- Patient T051 was lost to follow-up after the first trial visit (day 42). The site tried to contact this patient on several occasions without success.
- Patient U024 died after the third trial visit (day 180) due to high grade B-cell non Hodgkin lymphoma stage IV B and chronic obstructive pulmonary disease.

Table S3: Summary of participants and reasons for not attending at each time-point

	Baseline	Day 42	Day 90	Day 180	Day 210
**DNA**				1	
**Lost to follow-up**			1		
**Withdrawal**	1		1*		
**Deaths**					1

*withdrawal of trial treatment (patient T047)

The overall proportion of censored and missing data for this trial was small. Table S4 shows the proportion of cases excluded from the analyses for each outcome measure by treatment arm. Note that as explained in the Statistical Analysis Plan (above) and the methods section of the RAPS Trial manuscript, values below the lower limits of detection (LLOD) of the assays for the thrombin generation parameters and rivaroxaban levels (i.e. censored values) were excluded from the statistical analysis.

Table S4: Number and proportion of cases excluded from the analyses by outcome measure and treatment arm

	Rivaroxaban (N = 57)				Warfarin (N = 59)			
			Total excluded from the analysis				Total excluded from the analysis	
	Nc	Nm	N	%	Nc	Nm	N	%
ETP (nmol/L per minute) - baseline	3	0	3	5.3	1	0	1	1.7
ETP (nmol/L per minute) - day 42	0	0	0	0.0	2	1	3	5.1
Lag time (min) - baseline	3	0	3	5.3	1	0	1	1.7
Lag time (min) - day 42	1	0	1	1.8	1	1	2	3.4
Time to peak (min) - baseline	3	0	3	5.3	1	0	1	1.7
Time to peak (min) - day 42	0	0	0	0.0	1	1	2	3.4
Peak (nmol/L) - baseline	3	0	3	5.3	1	0	1	1.7
Peak (nmol/L) - day 42	0	0	0	0.0	2	1	3	5.1
Prothrombin fragment 1.2 (PF1.2) (pmol/L) - baseline	0	0	0	0.0	0	1	1	1.7
Prothrombin fragment 1.2 (PF1.2) (pmol/L) - day 42	0	0	0	0.0	0	1	1	1.7
Thrombin-antithrombin complex (TAT) (µg/L) - baseline	0	0	0	0.0	0	1	1	1.7
Thrombin-antithrombin complex (TAT) (µg/L) - day 42	0	0	0	0.0	0	1	1	1.7
D-dimer (mg/L FEU) - baseline	0	0	0	0.0	0	1	1	1.7
D-dimer (mg/L FEU) - day 42	0	0	0	0.0	0	1	1	1.7
Rivaroxaban anti-factor Xa (µg/L) - day 42	6	0	6	8.8	N/A	N/A	N/A	N/A
Factor X amidolytic (IU/dL) - day 42	N/A	N/A	N/A	N/A	0	1	1	1.7
TTR – Baseline*	N/A	2	2	3.5	N/A	7	7	11.9
Patients with 1 INR	N/A	1			N/A	2		
Patients with 2 INRs	N/A	1			N/A	5		
TTR – Day 180*	N/A		N/A		N/A	2	2	3.4
Patients with 0 INRs						2	2	
Patients with 1 INR	N/A		N/A		N/A	0	0	
Patients with 2 INRs	N/A		N/A		N/A	0	0	
EQ-5D-5L Health Utility - baseline	N/A	0	0	0	N/A	0	0	0
EQ-5D-5L Health Utility - day 180	N/A	1	1	1.8	N/A	4	4	6.8
EQ-5D-5L Health State: VAS –baseline	N/A	0	0	0.0	N/A	1	1	1.7
EQ-5D-5L Health State: VAS – day 180	N/A	1	1	1.8	N/A	3	3	5.1

*Patients with less than 3 INR readings were excluded from the analysis
Nc=Number of censored values (values above or below the limit of detection of the assay)
Nm=Number of missing values
N/A=not applicable

K. Treatment exposure

Table S5 shows the measures of treatment exposure for the 113 patients that completed the trial treatment visits (day 180).

Patient T047 allocated to warfarin withdrew from trial treatment after the patient's first trial visit (day 42) as his general practitioner (GP) offered to start him on rivaroxaban outside the trial. The patient did not attend the day 90 trial visit. However, he returned to the clinic for the day 180 assessment. The remaining 58 patients remained in their allocated warfarin treatment with 6/58 (10%) of them switching to rivaroxaban after trial completion.

Table S5: Measures of treatment exposure by treatment allocated and assessment time-point

		Up to day 180	Up to day 180
		**Rivaroxaban (N = 56)**	**Warfarin (N = 57)**
**Initial rivaroxaban dose, N (%)**	20 mg OD	54 (96.4)	N/A
	15 mg OD	2 (3.6)	N/A
**Patient remains on allocated treatment, N (%)**	Yes	56 (100.0)	56 (100.0)
	No	0 (0.0)	0 (0.0)
**Temporary interruption of allocated treatment, N (%)**	No	40 (71.4)	39 (68.4)
	Yes	16 (28.6)	17 (29.8)
**Reason for interrupting treatment, N**	Bleeding	1	1
	Bridging anticoagulation	1	4
	Illness	0	1
	Elevated ALT (IU/L)	1	0
	Alternative anticoagulant	1	0
	Surgery	1	0
	High INR readings	0	8
	1 or more missing doses	11	5
	Rivaroxaban offered by GP	0	0
	Patient's decision	0	1
**Discontinuation of allocated treatment, N (%)**	Yes	0 (0.0)	1 (1.8)
	No	56 (100.0)	56 (98.2)
**Reason for discontinuing treatment, N (%)**	Toxicity/SAE	0	0
	Clinical decision	0	0

	Patient request	0	1
	Renal impairment	0	0
	Pregnancy	0	0
	Bleeding	0	0
	Other	0	0

N/A=not applicable

The proportion of patients that interrupted their treatment at some stage during the trial was similar in both treatment arms. The most common reason for temporarily interrupting the treatment was the patients missing at least one dose of their medication.

The 3 patients who did not make it to Day 180 also remained in their allocated treatment for the time they were in the study, and only one of them temporarily interrupted her treatment (i.e. warfarin) due to an increased INR.

Note that reasons for interruptions have been collected as free text variables in the CRFs. Of the 33 patients that had their treatment interrupted at some stage during the trial, 27 (14 allocated to rivaroxaban and 13 to warfarin) answered “other” to the reason for interrupting treatment.

L. RAPS Trial Investigators

University College London Hospitals NHS Foundation Trust and University College London

Dr Hannah Cohen: Chief Investigator, Consultant Haematologist, Department of Haematology, University College London Hospitals NHS Foundation Trust and Honorary Reader, Haemostasis Research Unit, Department of Haematology, University College London, 3rd Floor West, 250 Euston Road, London, NW1 2PG, UK

Professor David A Isenberg: Principal Investigator, Consultant Rheumatologist, University College London Hospitals NHS Foundation Trust and Head of the Academic Department of Rheumatology, Centre for Rheumatology, Division of Medicine, University College London, 4th Floor, Rayne Building, 5 University Street, London WC1E 6JF, UK

Professor Anisur Rahman: Consultant Rheumatologist, University College London Hospitals NHS Foundation Trust and Professor of Rheumatology, Centre for Rheumatology, Division of Medicine, University College London, 4th Floor, Rayne Building, 5 University Street, London WC1E 6JF, UK

Dr Ian Giles: Consultant Rheumatologist, University College London Hospitals NHS Foundation Trust and Reader, Centre for Rheumatology, Division of Medicine, University College London, 4th Floor, Rayne Building, 5 University Street, London WC1E 6JF, UK

Dr John Ioannou: Consultant Rheumatologist, University College London Hospitals NHS Foundation Trust and Reader, Centre for Rheumatology, Division of Medicine, University College London, 4th Floor, Rayne Building, 5 University Street, London WC1E 6JF, UK.

University College London Haemostasis Research Unit

Professor Samuel J Machin: Emeritus Professor of Haematology, Haemostasis Research Unit, Department of Haematology, University College London, 1st Floor, 51 Chenies Mews, London, WC1E 6HX, UK

Dr Ian J Mackie: Laboratory Director, RAPS Central Laboratory and Reader, Haemostasis Research Unit, Department of Haematology, University College London, 1st Floor, 51 Chenies Mews, London, WC1E 6HX, UK

Dr Andrew Lawrie: Senior Research Associate, Haemostasis Research Unit, Department of Haematology, University College London, 1st Floor, 51 Chenies Mews, London, WC1E 6HX, UK

Dr Maria Efythymiou: Trial Post Doctoral Scientist, Haemostasis Research Unit, Department of Haematology, University College London, 1st Floor, 51 Chenies Mews, London, WC1E 6HX, UK

Dr Deepa RJ Arachchillage: Clinical Research Fellow, University College London Hospitals NHS Foundation Trust and Haemostasis Research Unit, Haematology Dept, University College London, 1st Floor, 51 Chenies Mews, London, WC1E 6HX, UK

University College London Comprehensive Clinical Trials Unit

Professor Caroline J Doré: Lead Trial Statistician, University College London Comprehensive Clinical Trials Unit, Gower Street, London, WC1 6BT, UK

Yvonne Sylvestre: Trial Statistician, University College London Comprehensive Clinical Trials Unit, Gower Street, London, WC1 6BT, UK

Dr Nicola Muirhead: Trial Clinical Project Manager, University College London Comprehensive Clinical Trials Unit, Gower Street, London WC1 6BT, UK

Simon Clawson: Trial Manager (to 17 April 2015), University College London Comprehensive Clinical Trials Unit, Gower Street, London WC1 6BT, UK

Heather Short: Trial Manager (from 20 April 2015), University College London Comprehensive Clinical Trials Unit, Gower Street, London WC1 6BT, UK

Susan Tebbs: Deputy Director, University College London Comprehensive Clinical Trials Unit, Gower Street, London WC1 6BT, UK

Ann Marie Swart: Director (to 13 May 2012), University College London Comprehensive Clinical Trials Unit, Gower Street, London WC1 6BT, UK

Guy's and St Thomas' Hospitals NHS Foundation Trust and Kings College London

Professor Munther Khamashta: Principal Investigator (to 01 April 2015), Consultant Physician, Guy's and St Thomas' NHS Foundation Trust (to 01 April 2015): Lupus Research Unit, Division of Women's Health, King's College London; Guy's and St Thomas' Hospitals NHS Foundation Trust, Westminster Bridge Road, London, SE1 7EH, UK

Professor Beverley J Hunt: Principal Investigator (from 02 April 2015), Consultant Haematologist, Department of Thrombosis and Haemophilia, Guy's and St Thomas' Hospitals NHS Foundation Trust; Department of Haematology, Kings College London; Guy's and St Thomas' Hospital, 4th Floor, North Wing, Westminster Bridge Road, London, SE1 7EH, UK

Dr Maria L Bertolaccini: Senior Research Associate, Lupus Research Unit Laboratory, Kings College London, Guys and St Thomas' NHS Foundation Trust, 4th Floor, North Wing, Westminster Bridge Road, London, SE1 7EH, UK; (from 01 January 2016): Academic Department of Vascular Surgery, Cardiovascular Division, Faculty of Life Sciences and Medicine, King's College London, 1st Floor North Wing, Westminster Bridge Road, St Thomas' Hospital, London, SE1 7EH

Professor David D'Cruz: Consultant Rheumatologist, Lupus Research Unit, Kings College London and Guy's and St Thomas' Hospitals NHS Foundation Trust, Westminster Bridge Road, London, SE1 7EH, UK

Dr Maria Cuadrado: Consultant Rheumatologist, Lupus Research Unit, Guy's and St Thomas' Hospitals NHS Foundation Trust, Westminster Bridge Road, London, SE1 7EH, UK

Dr Maria Ruiz-Castellano: Clinical Research Fellow, Lupus Research Unit, King's College London; Guy's and St Thomas' Hospitals NHS Foundation Trust, Westminster Bridge Road, London, SE1 7EH, UK

Dr Giovanni Sanna: Consultant Rheumatologist, Lupus Research Unit, Guy's and St Thomas' Hospitals NHS Foundation Trust, Westminster Bridge Road, London, SE1 7EH, UK

Research staff who assisted with patient recruitment, data collection, and data management

University College London Hospitals NHS Foundation Trust and University College London

Adepeju Adeokun: Research Nurse, Department of Haematology, University College London Hospitals NHS Foundation Trust and Honorary Reader, Haemostasis Research Unit, Department of Haematology, University College London, 1st Floor Central, 250 Euston Road, London, NW1 2PG, UK

Nyarko Ahwireng, Clinical Research Nurse, Department of Rheumatology, University College London Hospitals NHS Foundation, 250 Euston Road, London, NW1 2PG, UK

Krishna Hathi: Clinical Trials Practitioner, Non-Malignant Haematology, Cancer Clinical Trials Unit, University College London Hospitals NHS Foundation Trust, 250 Euston Road, London, NW1 2PG, UK

Sarah Taylor: Data Manager, Non-Malignant Haematology, Cancer Clinical Trials Unit, University College London Hospitals NHS Foundation Trust, 250 Euston Road, London, NW1 2PG, UK

Pajany Vythelingum: Clinical Research Nurse, Clinical Research Facility, University College Hospital, 25 Grafton Way, London WC1E 6DB

Guy's and St Thomas' Hospitals NHS Foundation Trust and Kings College London

Dr Karen Breen: Consultant: Department of Thrombosis and Haemophilia, Guy's and St Thomas' Hospitals NHS Foundation Trust; 4th Floor, North Wing, Westminster Bridge Road, London, SE1 7EH, UK

LeeMeng Choong: Clinical Research Nurse, Lupus Research Unit, King's College London; Guy's and St Thomas' Hospitals NHS Foundation Trust, Westminster Bridge Road, London, SE1 7EH, UK

Richard Glaves : Clinical Research Nurse, Department of Thrombosis and Haemophilia, Guy's and St Thomas' Hospitals NHS Foundation Trust; 4th Floor, North Wing, Westminster Bridge Road, London, SE1 7EH, UK

Louise Nel: Clinical Research Nurse, Lupus Research Unit, King's College London; Guy's and St Thomas' Hospitals NHS Foundation Trust, Westminster Bridge Road, London, SE1 7EH, UK

Dr Savino Sciascia: Clinical Research Fellow, Thrombosis & Lupus Research Unit, King's College London; Guy's and St Thomas' Hospitals NHS Foundation Trust, Westminster Bridge Road, London, SE1 7EH, UK

Johanna Young: Clinical Research Nurse, Department of Thrombosis and Haemophilia, Guy's and St Thomas' Hospitals NHS Foundation Trust; 4th Floor, North Wing, Westminster Bridge Road, London, SE1 7EH, UK

M. References

1. Cheung K, Oemar M, Oppe M, Rabin R. EQ-5D user guide. Basic information on how to use EQ-5D. Rotterdam: EuroQol Group;2009.

2. Van Hout B, Janssen MF, Feng YS et al. Interim scoring for the EQ-5D-5L: Mapping the EQ-5D-5L to EQ-5D-3L value sets. Value in Health 2012;**15**:708-15.

3. Rosendaal FR, Cannegieter SC, van der Meer et al. A method to determine the optimal intensity of oral anticoagulant therapy. Thromb Haemost 1993;**69**:236-9.

4. Dargaud Y, Luddington R, Gray E et al. Standardisation of thrombin generation test – which reference plasma for TGT?: An international multicentre study. Thromb Res 2010;**125**:353-6.

5. Brodin E, Seljeflot, Arnesen H, Hurlen M, Applebom H, Hansen JB. Endogenous thrombin potential (ETP) in plasma from patients with AMI during antithrombotic treatment. Thromb Res 2009;**123**:573–9.

6. Julious SA. Tutorial in biostatistics. Sample sizes for clinical trials with normal data. Stat Med 2004;**23**:1921-86.

7. ICH E9: International conference on harmonisation of technical requirements for registration of pharmaceuticals for human use: ICH harmonised tripartite guideline: Statistical principles for clinical trials. 1998. http://www.ich.org/products/guidelines/efficacy/efficacy-single/article/statistical-principles-for-clinical-trials.html.

   Addendum 2014: http://www.ich.org/products/guidelines/efficacy/efficacy-single/article/addendum-statistical-principles-for-clinical-trials.html

8. White IR, Royston P, Wood AM. Multiple imputation using chained equations: Issues and guidance for practice. Stat Med 2011;**30**:377-99.

9. ICH E3: International conference on harmonisation of technical requirements for registration of pharmaceuticals for human use: ICH harmonised tripartite guideline: structure and content of clinical study reports. 1995. http://www.ich.org/products/guidelines/efficacy/efficacy-single/article/structure-and-content-of-clinical-study-reports.html

10. Pengo V, Tripodi A, Reber G et al. Update of the guidelines for lupus anticoagulant detection. Subcommittee on Lupus Anticoagulant/Antiphospholipid Antibody of the Scientific and Standardisation Committee of the International Society on Thrombosis and Haemostasis. J Thromb Haemost 2009;**7**:1737-40.

11. Miyakis S, Lockshin MD, Atsumi T et al. International consensus statement on an update of the classification criteria for definite antiphospholipid syndrome (APS). J Thromb Haemost 2006;**4**:295-306.

12. Keeling D, Mackie I, Moore GW, Greer IA, Greaves M. British Committee for Standards in Haematology. Guidelines on the investigation and management of antiphospholipid syndrome. Br J Haematol 2012;**157**;47-58.
